# The Effect of Hyperoxemia on Neurological Outcomes of Adult Patients: A Systematic Review and Meta-Analysis

**DOI:** 10.1007/s12028-021-01423-w

**Published:** 2022-01-31

**Authors:** Chanawee Hirunpattarasilp, Hiroko Shiina, Nat Na-Ek, David Attwell

**Affiliations:** 1 Department of Neuroscience, Physiology, and Pharmacology, University College London, Gower Street, London, WC1E 6BT England, UK; 2 Princess Srisavangavadhana College of Medicine, Chulabhorn Royal Academy, 906 Kamphaeng Phet 6 Rd, Talat Bang Khen, Lak Si, Bangkok, 10210 Thailand; 3 Division of Pharmacy Practice, Department of Pharmaceutical Care, School of Pharmaceutical Sciences, University of Phayao, 19 Moo 2 Tambon Maeka Amphur Muang Phayao, Mae Ka, 56000 Thailand

**Keywords:** Hyperoxemia, Meta-analysis, Neurological outcome, Observational studies, Oxygen, Subarachnoid hemorrhage

## Abstract

Hyperoxemia commonly occurs in clinical practice and is often left untreated. Many studies have shown increased mortality in patients with hyperoxemia, but data on neurological outcome in these patients are conflicting, despite worsened neurological outcome found in preclinical studies. To investigate the association between hyperoxemia and neurological outcome in adult patients, we performed a systematic review and meta-analysis of observational studies. We searched MEDLINE, Embase, Scopus, Web of Science, Cumulative Index to Nursing and Allied Health Literature, and ClinicalTrials.gov from inception to May 2020 for observational studies correlating arterial oxygen partial pressure (PaO_2_) with neurological status in adults hospitalized with acute conditions. Studies of chronic pulmonary disease or hyperbaric oxygenation were excluded. Relative risks (RRs) were pooled at the study level by using a random-effects model to compare the risk of poor neurological outcome in patients with hyperoxemia and patients without hyperoxemia. Sensitivity and subgroup analyses and assessments of publication bias and risk of bias were performed. Maximum and mean PaO_2_ in patients with favorable and unfavorable outcomes were compared using standardized mean difference (SMD). Of 6255 records screened, 32 studies were analyzed. Overall, hyperoxemia was significantly associated with an increased risk of poor neurological outcome (RR 1.13, 95% confidence interval [CI] 1.05–1.23, statistical heterogeneity *I*^2^ 58.8%, 22 studies). The results were robust across sensitivity analyses. Patients with unfavorable outcome also showed a significantly higher maximum PaO_2_ (SMD 0.17, 95% CI 0.04–0.30, *I*^2^ 78.4%, 15 studies) and mean PaO_2_ (SMD 0.25, 95% CI 0.04–0.45, *I*^2^ 91.0%, 13 studies). These associations were pronounced in patients with subarachnoid hemorrhage (RR 1.34, 95% CI 1.14–1.56) and ischemic stroke (RR 1.41, 95% CI 1.14–1.74), but not in patients with cardiac arrest, traumatic brain injury, or following cardiopulmonary bypass. Hyperoxemia is associated with poor neurological outcome, especially in patients with subarachnoid hemorrhage and ischemic stroke. Although our study cannot establish causality, PaO_2_ should be monitored closely because hyperoxemia may be associated with worsened patient outcome and consequently affect the patient’s quality of life.

**Supplementary Information:**

The online version contains supplementary material available at 10.1007/s12028-021-01423-w.

## Introduction

Oxygen is frequently prescribed for the prevention and/or treatment of hypoxemia and tissue hypoxia [1]. However, giving too much oxygen causes hyperoxemia (arterial hyperoxia), for which a definition based on a partial pressure of arterial oxygen (PaO_2_) value has not been rigorously provided, although normoxemia is defined as a PaO_2_ of 80–100 mm Hg [2]. Although hyperoxemia has been linked to deleterious effects such as systemic vasoconstriction, increased oxidative stress [3, 4], and increased mortality [5–7], hyperoxemia is still common in general wards [8, 9] and intensive care units (ICUs) [10–14]. In recent studies, a PaO_2_ of 300 mm Hg or more was reported in 11–26% of the patients [15–19] and 46% of PaO_2_ measurements were hyperoxic [12] (defined as PaO_2_ > 110 mm Hg in that study). Unfortunately, hyperoxemia is sometimes left uncorrected [2, 12, 14]. This might reflect ICU culture, precautionary oxygen use, limited opportunities for quality improvement and revision of outdated practices (such as unnecessary oxygen supplementation), and the lack of a formal process for oxygen titration [10, 14, 20, 21].

Aside from preventing mortality, preserving neurological function is an important goal of critical care. Cerebral dysfunction causes morbidity and disability in patients, reducing patients’ quality of life and conferring an enormous socioeconomic burden on patients, their families, and society [22]. Worryingly, in preclinical studies, a high oxygen level is associated with worsened neurological outcome [23]. Because the results for neurological outcome from human studies are inconsistent, a synthesis of all available data is needed, especially because hyperoxemia is a potential modifiable factor related to a neurological outcome that can be easily monitored and treated.

## Methods

To explore how hyperoxemia correlates with neurological status, we conducted a systematic review and meta-analysis of published observational studies. Our primary objective was to compare neurological outcome in patients with hyperoxemia and patients without hyperoxemia. The secondary objective was to compare the levels of PaO_2_ in patients with poor and good outcomes.

We followed the Preferred Reporting Items for Systematic Reviews and Meta-Analyses (PRISMA) guidelines (Supplementary file 7: Additional File 1) and registered the study with the PROSPERO database (CRD42020187940). The protocol was edited once during title and abstract screening and was updated in PROSPERO accordingly.

### Literature Search

Studies were identified through searching the following databases: MEDLINE (Ovid; 1946 to the present), Embase (Ovid; 1947 to the present), Scopus (2004 to the present), Web of Science (1900 to the present), Cumulative Index to Nursing and Allied Health Literature (1937 to the present), and ClinicalTrials.gov (2000 to the present). The search was not restricted to specific publication types or languages provided if there was an abstract in English. We used search key words related to “hyperoxemia,” “hyperoxia,” “high oxygen,” “neurological outcome/disability,” and “human.” The search strategy (Supplementary file 7: Additional File 2) was reviewed by a librarian at University College London. The last search was on May 24th, 2020. Additional studies were discovered by searching systematic reviews, reference lists of articles, and unpublished studies on bioRxiv to identify all relevant works and minimize publication bias.

### Inclusion and Exclusion Criteria

We included all observational studies (both prospective and retrospective cohort studies and case control studies) investigating the effect of arterial hyperoxia on neurological outcome, which met the following eligibility criteria: (1) patients were hospitalized with acute medical or surgical conditions and (2) participants were adults (of any sex).

We limited our search to observational studies for the following reasons: the main outcome of interest was neurological outcome, which is seldom reported in clinical trials on hyperoxia; no clinical trials have studied the effect of hyperoxemia in certain diseases, e.g., subarachnoid hemorrhage (SAH); and observational studies provide data on patients with high levels of PaO_2_, to which deliberately exposing patients might be unethical in trials.

In each included study, a PaO_2_ cutoff value defined by that study was used to categorize patients into hyperoxemia and control groups. Our meta-analysis adopted this definition of hyperoxemia from each study, regardless of the PaO_2_ cutoff value and the qualifying time period, despite a certain level of clinical and methodological heterogeneity. This is because we could only compile data at study levels by pooling summary statistics from each study (this approach has been used in previous meta-analyses on hyperoxia) [5, 24, 25]. The fact that individual patient data from included studies were not available prevented us from employing a more consistent hyperoxemia definition.

We only analyzed studies defining hyperoxemia in terms of PaO_2_ for the following reasons: combining studies with different definitions of hyperoxemia (e.g., PaO_2_, oxygen saturation, fraction of inspired oxygen, or conservative/liberal oxygenation) introduces further methodological heterogeneity; defining oxygen excess in the body is difficult using oxygen saturation, which saturates at a PaO_2_ of 100 mm Hg, or fraction of inspired oxygen or oxygenation strategies, which do not directly measure body oxygen; and using absolute values of PaO_2_ allows further analyses, e.g., PaO_2_ subgroup analysis, meta-analyses of association strength from linear regressions, and quantification of PaO_2_ threshold that best differentiates patients with poor and good neurological outcomes.

Neurological prognoses were defined as functioning and disability of the nervous system affecting the following: body functions and structures and/or activities and participation [22]. We extracted outcome data from the longest follow-up period in each study.

We excluded studies if the study population was from patients with chronic pulmonary disease or receiving hyperbaric oxygenation. This was because patients with chronic lung diseases are at risk of oxygen-induced hypercapnia, which might independently affect outcomes [26], and hyperbaric oxygenation might exert additional effects associated with high pressure alone [1]. Studies reporting mortality without neurological outcomes were also excluded.

### Study Selection

Identified articles were independently screened as titles and abstracts by two reviewers (CH, HS) and then as a full text. Any disagreements were resolved by discussion and by a third reviewer (DA). We measured interrater agreement and Cohen’s kappa [27]. Adequate agreement was defined as percent agreement > 80% and Cohen’s kappa > 0.60 [27].

### Data Extraction

A data extraction sheet was developed, pilot tested, and then modified to finalize the form to extract relevant information (Supplementary file 7: Additional File 3). Data extraction was performed independently by two reviewers (CH, HS), and disagreements were resolved by discussion or a third author (DA). Studies with potentially overlapping populations were checked by comparing study characteristics and were confirmed with the authors. For multiple studies from the same group of patients, we only included articles with the lowest risk of bias or the largest number of patients if a similar risk of bias was found. Values that were not reported in the original article were estimated from graphs, when possible.

In articles using multiple thresholds for hyperoxemia, we used data from the group with the most extreme PaO_2_ level. If control groups were not defined (e.g., Janz et al. [28] separated patients into PaO_2_ quartiles without stating which quartile was the control group), we combined all nonhyperoxemia groups into one control group. When neurological outcomes were evaluated using multiple assessment scales, the scale with the largest patient population was selected. For studies reporting raw scores on neurological outcome scales, we dichotomized the scores into poor and good outcomes employing commonly used cutoff points (Cerebral Performance Category [CPC] score ≥ 3, Glasgow Outcome Scale [GOS] ≤ 3, and Glasgow Outcome Scale extended [GOSE] ≤ 4 for poor prognoses).

Studies were grouped by patient ventilation status into the following categories: (1) definitely ventilated, all patients were noted to be on a ventilator or oxygenator; (2) probably ventilated, patients likely required ventilation, such as patients with trauma with GCS ≤ 8 [29] and comatose patients following the return of spontaneous circulation (ROSC) undergoing targeted temperature management, but for whom ventilation status was not mentioned; (3) ventilated and nonventilated, patients who were either mechanically ventilated or not ventilated; and (4) unassessable, no ventilation status was noted and the indication for giving ventilation was unclear. Seven authors responded to requests for data confirmation and provided additional data.

### Risk of Bias Assessment

Included studies were assessed for bias using the Newcastle–Ottawa Scale (NOS) for assessing the quality of cohort and case control studies. Studies were classified as “good” quality if the score in each NOS domain was > 0 and the total score was > 6/9.

Assessment was independently performed by two reviewers (CH, HS) in duplicate. Any disagreements were resolved by discussion. No studies were excluded based on bias assessment. However, for the sensitivity analysis only good quality studies were included.

### Statistical Analysis

For comparing neurological outcomes in patients with hyperoxemia and control patients, we calculated unadjusted relative risks (RRs) and 95% confidence intervals (95% CIs) from the number of cases (with poor neurological outcome) and noncases (without poor neurological outcome) in hyperoxemia and control groups.

We pooled study estimates using the inverse variance method for a fixed-effect model if there was no significant heterogeneity among studies. However, a random-effects model was applied if significant clinical or statistical heterogeneity was found. Statistical heterogeneity was measured using the Cochrane Q statistic, which assesses whether differences among the studies were due to chance, and we used the *I*^2^ test to quantify inconsistency across studies. A *χ*^2^ test *p* value < 0.1 or an *I*^2^ value > 75% was used to define significant statistical heterogeneity [30].

Publication bias was evaluated from the symmetry of a contour-enhanced funnel plot of RR (on a natural log scale) against the inverse standard error of the natural log of RR, and performing Egger’s test [30]. We applied the trim and fill method when the funnel plot showed an asymmetry or a *p* value from Egger’s test was less than 0.05, to make the funnel plot more symmetrical, enabling the computation of hypothetical results [30].

Prespecified sensitivity analyses included using unadjusted odds ratio (OR) as an effect size, analyzing only good quality publications, and analyzing after discarding sources of heterogeneity. Moreover, we performed sensitivity analyses using the extracted adjusted RR and adjusted OR, for which other confounders had been corrected. Adjusted OR was converted to adjusted RR, and vice versa, as previously reported [31]. Predetermined subgroup analysis was carried out according to patients’ underlying diseases. Post hoc subgroup analyses were performed based on the following: functional outcomes; exclusion of hypoxemia in controls; PaO_2_ level used to define hyperoxemia, grouping studies into those with PaO_2_ cutoff values ≥ 300 mm Hg, between 200 and 299 mm Hg, and between 100 and 199 mm Hg; and ventilation status. Additional post hoc analyses were performed to examine the correlation between oxygenation level and poor neurological outcome (Supplementary file 7: Additional File 4), and to determine a PaO_2_ threshold value that best differentiated favorable and unfavorable outcome groups (Supplementary file 7: Additional File 5).

For the secondary objective (comparing PaO_2_ in patients with poor and good outcomes), we calculated the pooled Hedge’s g parameter and its 95% CI to compare PaO_2_ in patients with poor and good neurological status. We separately analyzed the maximum and mean PaO_2_ from both groups. If multiple blood gas analyses were performed for each patient, the summary statistics used in the original papers (means/medians) calculated from the highest PaO_2_ and average PaO_2_ of each patient were used in the pooled analysis of maximum PaO_2_ and mean PaO_2_. We used formulas [32, 33] to estimate means and standard deviations (SDs) from studies reporting median and interquartile ranges (IQRs) of PaO_2_.

All analyses were performed at a study level using STATA program version 15 (IC version, StataCorp) and R program version 4.0.2.

## Results

### Search Results

**Fig. 1:**
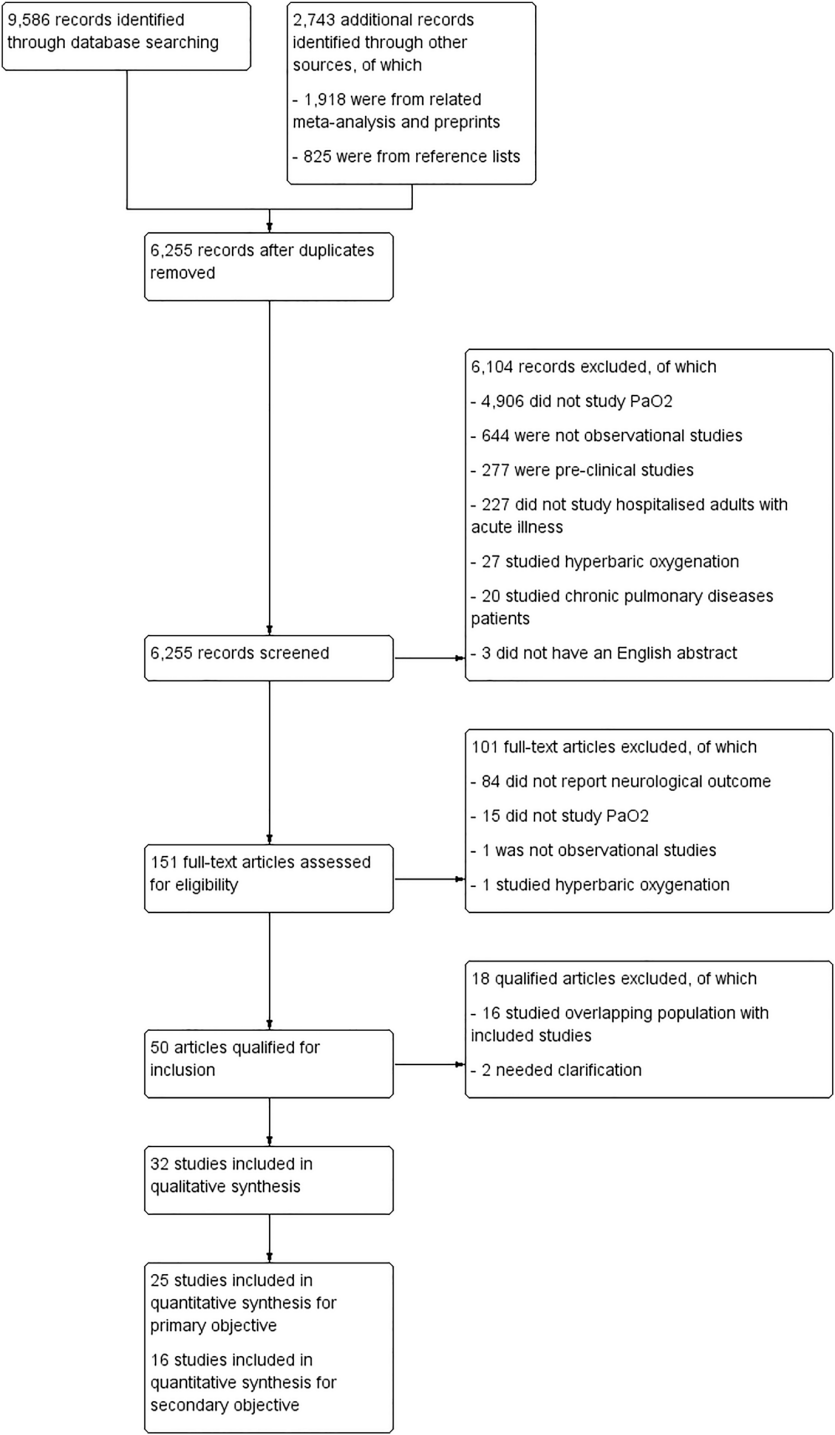
Study flow diagram. PaO_2_, arterial oxygen partial pressure

We identified 9586 and 2743 records from database searching and other sources, respectively. After removing duplicates, 6255 records were screened through titles and abstracts. Of these, 6104 citations were discarded for not meeting the eligibility criteria, leaving 151 for full text screening, of which 101 records were excluded for failing to meet the criteria. These included studies assessing outcomes other than neurological outcome [34–37]. Fifty records were eligible for inclusion; however, 18 were discarded due to overlapping patient populations and missing critical information (Supplementary file 7: Additional File 6). Finally, we included 32 quantitative studies, of which 25 and 16 studies were included in the meta-analysis for our primary and secondary objectives, respectively. Figure 1 shows the study flow diagram. Interrater agreement was 87% and Cohen’s kappa was 0.71.

### Study Characteristics

All 32 selected studies were cohort studies published in English from 2011 to 2020, of which 11 were multicenter studies and 21 were single-center studies (Table 1). Twenty-six studies were full publications and six were conference abstracts.

**Table 1:** Study characteristics

Study	Study design/study settings	Population	Principle diagnosis/additional data	Ventilation status	Type of PaO_2_	Hyperoxemia/controls, PaO_2_ (mm Hg)	Outcome measures/timing	Scores for poor outcome
Alali [15]	CR/multicenter	417	TBI	Probably ventilated	Average	> 350^a^/ < 350^a^	GOSE/6 months	1–4^b^
Bolduc [57]	CR/single-center	265	Cardiac arrest/TTM	Unassessable	N/A	≥ 300/N/A	CPC/N/A	3–5
Brenner [58]	CR/single-center	1547	TBI	Unassessable	Average	> 200/100–200	GCS/Discharge	3–8
Chang [17]	CR/single-center	291	Cardiac arrest/ECMO	Definitely ventilated	First	≥ 300/60–300	CPC/Discharge	3–5
Ebner [59]	CR/multicenter	869	Cardiac arrest/OHCA, TTM	Probably ventilated	Highest	> 300/60–300	CPC/6 months	3–5
Elmer [60]	CR/single-center	184	Cardiac arrest	Definitely ventilated	At specific time	N/A/N/A	CPC/Discharge	N/A
Fujita [61]	CR/multicenter	129	TBI/TTM	Probably ventilated	First	N/A/N/A	GOS/6 months	1–3
Fukuda [46]	CR/single-center	197	SAH	Ventilated and nonventilated	Average	≥ 250^a^/ < 250^a^	GOS/Discharge	1–3
Gaieski [62]	CR/multicenter	111	Cardiac arrest/TTM	Probably ventilated	N/A	> 300/N/A	N/A/N/A	N/A
Humaloja [16]	CR/single-center	1110	Cardiac arrest	Unassessable	First	> 300^a^ /60–120^a^	CPC/1 year	3–5
Janz [28]	CR/single-center	170	Cardiac arrest/TTM	Probably ventilated	Highest	310–608^a^/ ≤ 310^a^	CPC/Discharge	3–5
Jeon [63]	CR/single-center	202	SAH	Definitely ventilated	Average	≥ 173/ < 173^a^	mRS/3 months	4–6
Johnson [64]	CR/multicenter	544	Cardiac arrest	Unassessable	At specific time	> 300/60–300	CPC/Discharge	3–5
Kiguchi [39]	CR/multicenter	662	Cardiac arrest/OHCA	Unassessable	First	≥ 300/ < 300	CPC/1 months	3–5
Kupiec [65]	CR/single-center	93	post-CPB	Definitely ventilated	Highest	≥ 200/120–200	POD/3 d	+
Lång [47]	CR/multicenter	432	SAH	Definitely ventilated	Average	> 150/97.5–150	GOS/3 months	1–3
Lee [66]	CR/single-center	213	Cardiac arrest/TTM	Probably ventilated	Average	≥ 156.7^a^/116.9–134.9^a^	CPC/Discharge	3–5
Li [67]	CR/single-center	244	SAH	Unassessable	Highest	> 200/ ≤ 200	GOS/3 months	1–3
Lopez [68]	CP/single-center	333	Ischemic stroke/IAMT	Definitely ventilated	Highest	> 120/ ≤ 120	mRS/3 months	4–6
Oh [69]	CR/multicenter	792	Cardiac arrest/IHCA	Unassessable	At specific time	≥ 300/60–299	CPC/Discharge	3–5
Peluso [18]	CR/single-center	356	Cardiac arrest/TTM	Definitely ventilated	Highest	> 300/ ≤ 300	CPC/3 months	3–5
Popovic [38]	CR/single-center	49	TBI	Definitely ventilated	First	> 200/100–200	GOS/Discharge	1–3^b^
Rai [70]	CR/single-center	88	Cardiac arrest/TTM	Probably ventilated	N/A	≥ 300/60–299	N/A/N/A	N/A
Roberts [71]	CP/multicenter	280	Cardiac arrest/TTM	Definitely ventilated	Highest	> 300/ ≤ 300	mRS/Discharge	4–6
Russell [72]	CR/single-center	471	Traumatic injuries	Definitely ventilated	Highest	N/A/N/A	GCS/Discharge	N/A
Sadaka [73]	CR/single-center	165	TBI	Unassessable	First	≥ 245/60–240	GOS/Discharge	1–3
Sadaka [74]	CR/single-center	56	Cardiac arrest/TTM	Probably ventilated	First	≥ 250/60–249	CPC/Discharge	3–5
Spindelboeck [75]	CR/multicenter	145	Cardiac arrest/OHCA	Definitely ventilated	First	> 300/61–300	CPC/1 months or discharge	3–5
Vaahersalo [40]	CP/multicenter	409	Cardiac arrest/OHCA	Definitely ventilated	Average	128–237^a^/ < 128^a^	CPC/12 months	3–5
Wang [76]	CR/single-center	550	Cardiac arrest/IHCA	Definitely ventilated	First	> 300/60–300	CPC/Discharge	3–5
Yokoyama [19]	CR/single-center	196	SAH	Definitely ventilated	Highest	> 300^a^/60–120	mRS/Discharge	3–6
Youn [77]	CR/single-center	187	Cardiac arrest/OHCA, TTM	Probably ventilated	Area under curve	N/A/N/A	CPC/6 months	3–5

CA, cardiac arrest, CP, prospective cohort, CPB, cardiopulmonary bypass, CPC, cerebral performance category, CPR, cardiopulmonary resuscitation, CR, retrospective cohort, DCI, delayed cerebral ischemia, ECMO, extracorporeal membrane oxygenation, GCS, Glasgow Coma Scale, GOS, Glasgow Outcome Scale, GOSE, Glasgow Outcome Scale extended, IAMT, intraarterial mechanical thrombectomy, IHCA, in-hospital cardiac arrest, mRS, modified Rankin Scale, N/A, not applicable, OHCA, out-of-hospital cardiac arrest, PaO_2_, arterial partial pressure of oxygen, POD, postoperative delirium, ROSC, return of spontaneous circulation, SAH, subarachnoid hemorrhage, TBI, traumatic brain injury, TTM, targeted temperature management

^a^Represents groups assigned by the reviewers using most extreme value for PaO_2_ and the largest number of participants for neurological outcome

^b^Represents groups assigned by the reviewers using commonly used cutoff points

### Participants

The total number of participants from all included studies was 11,757. There were 7282 patients who experienced cardiac arrest (CA) (19 studies), 2307 patients with traumatic brain injury (TBI) (five studies), 1271 patients with SAH (five studies), 471 patients with general traumatic injury (one study), 333 patients with ischemic stroke (one study), and 93 patients who had post cardiopulmonary bypass (CPB) surgery (one study).

### Exposure

Oxygenation level was defined using the first measured PaO_2_ in nine studies, the highest PaO_2_ in nine studies, the average PaO_2_ in seven studies, PaO_2_ at a specific time in three studies and the area under the plot of PaO_2_ against time in one study. The remaining three studies did not specify which PaO_2_ values were used. The timing of PaO_2_ assessments varied from time of admission to six days after admission; however, the majority of studies measured PaO_2_ within the first 24 h (16 studies). Twenty-eight studies systematically categorized hyperoxemia groups based on their PaO_2_ values. Of these, 24 clearly stated hyperoxemia groups. However, four categorized hyperoxemia using different classes: tertiles, quartiles, or multiple PaO_2_ levels, and thus the highest PaO_2_ levels were assigned as hyperoxemia. To be defined as hyperoxemia, the PaO_2_ threshold values ranged from 120 to 350 mm Hg with most studies using 300 mm Hg as their threshold (15 studies).

### Nonexposure

Nonexposure was defined as PaO_2_ levels below the thresholds employed, which was categorized as normoxemia (excluding hypoxemia) in 16 studies, and as nonhyperoxemia (including hypoxemia) in ten studies.

### Outcomes

Neurological outcomes were assessed using the CPC in 16 studies, GOS in six studies, mRS in four studies, GCS in two studies, GOSE in one study, presence of postoperative delirium (POD) in one study, and undefined scoring systems in two studies. Thirty studies dichotomized neurological outcomes as poor or good, and poor outcomes were defined using CPC ≥ 3 in 15 studies, GOS ≤ 3 in six studies, mRS ≥ 4 in three studies, mRS ≥ 3 in one study, GCS ≤ 8 in one study, GOSE ≤ 4 in one study, and having POD in one study (no information was given for two studies). Two studies [15, 38] reported raw ordinal scores so dichotomization was performed by reviewers. Furthermore, timing of outcome assessments varied from time of hospital discharge up to 12 months post discharge. For the secondary objective, 16 studies reported PaO_2_ values in poor and good neurological outcome groups. Statistical measures calculated from the maximum and mean PaO_2_ values for each patient in case of multiple blood gas analyses, and a single value of PaO_2_ in case of single blood gas analysis, were reported in 11, nine, and four studies, respectively. Of these, ten studies reported the mean and SD, and six reported the median and IQR.

### Risk of Bias Within Studies

According to the NOS, ten studies (31%) were good quality studies (Table 2).

**Table 2:** Study quality assessment based on NOS

Study	Selection (4)				Comparability (2)		Outcome (3)			Risk of bias
	1.1 Representat-iveness of the exposed cohort	1.2 Selection of the nonexposed cohort	1.3 Ascertain-ment of exposure	1.4 Demonstration that outcome of interest was not present at start of study	2.1 Controlled for main factors (age and gender)	2.2. Controlled for additional factors (baseline neurologic status)	3.1 Assessment of outcome	3.2 Was follow-up long enough for outcomes to occur	3.3 Adequacy of follow-up of cohorts	
Alali [15]	1	1	1	0	1	0	1	1	0	Not good
Bolduc [57]	N/A	N/A	N/A	N/A	N/A	N/A	N/A	N/A	N/A	N/A
Brenner [58]	1	1	1	0	1	1	1	1	0	Good
Chang [17]	1	1	1	0	1	1	1	0	1	Good
Ebner [59]	1	1	1	0	1	1	1	1	1	Good
Elmer [60]	1	1	1	0	1	1	1	0	1	Good
Fujita [61]	1	1	1	0	1	1	1	1	0	Good
Fukuda [46]	1	1	1	0	1	1	1	0	0	Not good
Gaieski [62]	N/A	N/A	N/A	N/A	N/A	N/A	N/A	N/A	N/A	N/A
Humaloja [16]	1	1	1	0	1	1	1	1	1	Good
Janz [28]	1	1	1	0	1	0	1	0	1	Not good
Jeon [63]	1	1	1	0	1	1	1	1	0	Good
Johnson [64]	1	1	1	0	1	0	1	0	0	Not good
Kiguchi [39]	N/A	N/A	N/A	N/A	N/A	N/A	N/A	N/A	N/A	N/A
Kupiec [65]	1	1	1	1	0	0	1	1	0	Not good
Lång [47]	1	1	1	0	0	0	1	1	1	Not good
Lee [66]	1	1	1	0	1	0	1	0	0	Not good
Li [67]	1	1	1	0	1	1	1	1	0	Good
Lopez [68]	1	1	1	0	1	1	1	1	0	Good
Oh [69]	1	1	1	0	1	0	1	0	0	Not good
Peluso [18]	1	1	1	0	1	0	1	0	1	Not good
Popovic [38]	1	1	1	0	0	0	1	0	0	Not good
Rai [70]	N/A	N/A	N/A	N/A	N/A	N/A	N/A	N/A	N/A	N/A
Roberts [71]	1	1	1	0	1	0	1	0	0	Not good
Russell [72]	1	1	1	0	1	0	1	1	0	Not good
Sadaka [73]	N/A	N/A	N/A	N/A	N/A	N/A	N/A	N/A	N/A	N/A
Sadaka [74]	N/A	N/A	N/A	N/A	N/A	N/A	N/A	N/A	N/A	N/A
Spindelboeck [75]	1	1	1	0	1	0	1	0	0	Not good
Vaahersalo [40]	1	1	1	0	1	0	1	1	0	Not good
Wang [76]	1	1	1	1	1	1	1	0	1	Good
Yokoyama [19]	1	1	1	0	1	1	1	0	0	Not good
Youn [77]	1	1	1	0	1	0	1	1	0	Not good

N/A, not applicable, NOS, Newcastle–Ottawa Scale

### Quantitative Data Synthesis

#### Primary Objective

**Fig. 2:**
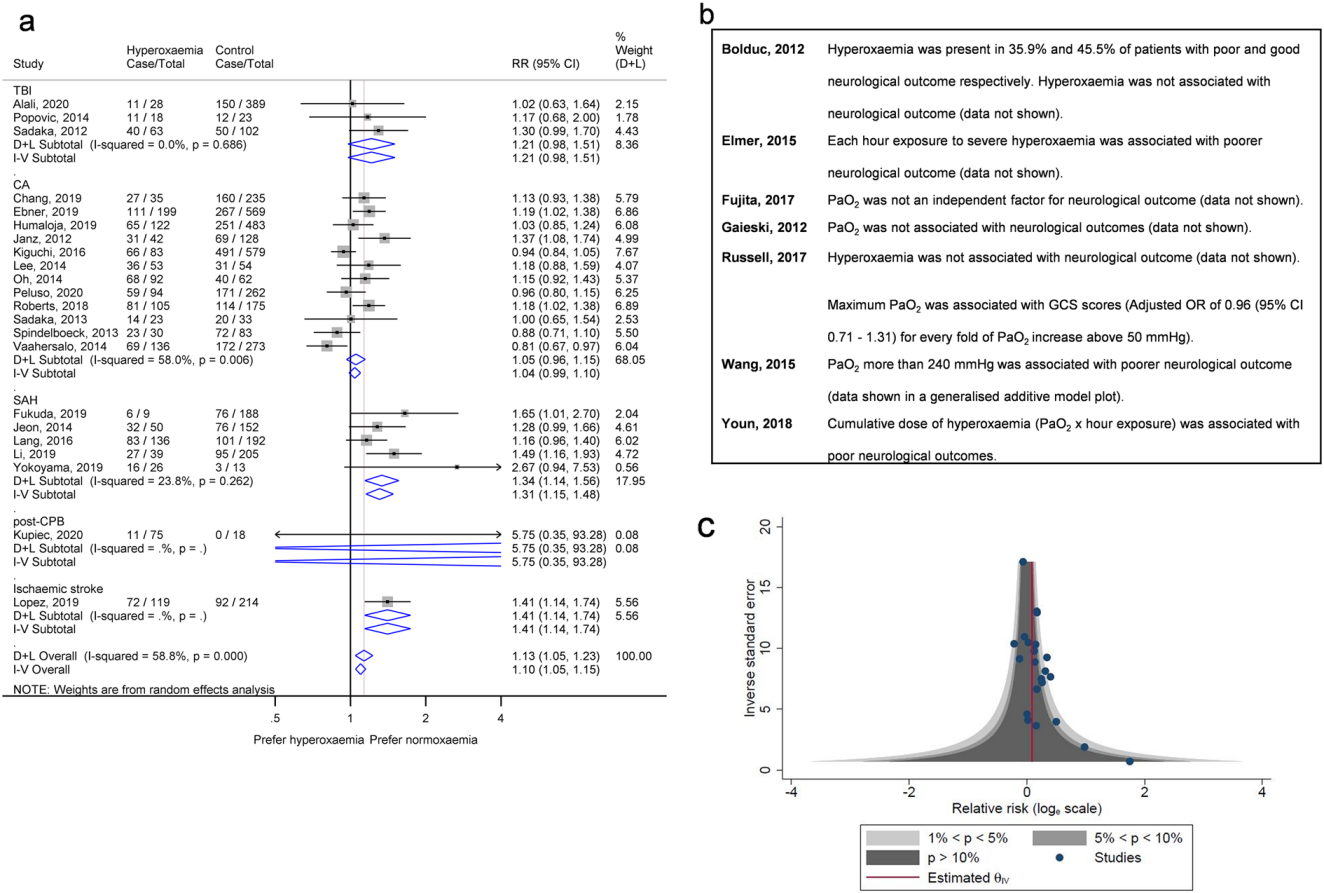
Main analysis of the first objective showing association of hyperoxemia and poor neurological outcomes. **a** Forest plot of unadjusted RRs of poor neurological outcome. The boxes show the effect estimates from the individual studies and the diamonds represent pooled results in each subgroup and overall analysis. The length of horizontal lines across the boxes and the width of the diamonds illustrates the 95% CI. The gray vertical line at one is the line of null effect, and the red vertical line shows the pooled effect estimate of the whole analysis. **b** Results of studies that were not included in the meta-analysis because of no information on study estimates and different definitions of high oxygen group (e.g., time spent exposed to hyperoxemia). **c** Contour-enhanced funnel plot for main analysis. CI, confidence interval, GCS, Glasgow Coma Scale, PaO_2_, arterial oxygen partial pressure, RR, relative risk, TBI, traumatic brain injury

Twenty-five studies provided numerical data on study measures. Of those, 22 studies were included in the meta-analysis for unadjusted RR, providing a population of 6009 participants with 3473 having poor neurological outcome. Hyperoxemia was significantly associated with poor neurological outcome (RR 1.13, 95% CI 1.05–1.23, *p* = 0.002, using a random-effects model) with significant heterogeneity among studies (*I*^2^ 58.8%, *p* < 0.001; Fig. 2a). Results from studies which were not included in the meta-analysis are summarized in Fig. 2b.

A contour-enhanced funnel plot was asymmetrical, suggesting the presence of publication bias [30] and Egger’s test showed a significant result (*p* = 0.034; Fig. 2c). Even after applying the trim and fill method [30], hyperoxemia was significantly associated with poor neurological outcome (RR 1.12, 95% CI 1.03–1.21, *p* = 0.007; Table 3).

**Table 3:** Summary of subgroup analysis according to principal diagnosis

Analysis	First objective				Secondary objective	
	Unadjusted effect size		Adjusted effect size		SMD	
	RR	OR	RR	OR	Maximum PaO_2_	Mean PaO_2_
Overall	22 studies	22 studies	11 studies	12 studies	15 studies	13 studies
Random-effects	1.13 (1.05, 1.23)*	1.37 (1.11, 1.68)*	1.26 (1.12, 1.41)*	1.55 (1.21, 1.99)*	0.17 (0.04, 0.30)*	0.25 (0.04, 0.45)*
Fixed-effect	1.10 (1.05, 1.15)*	1.30 (1.14, 1.48)*	1.26 (1.18, 1.34)*	1.49 (1.27, 1.74)*	0.14 (0.08, 0.19)*	0.16 (0.10, 0.21)*
Trim and fill method	1.12 (1.03, 1.21)*	1.31 (1.07, 1.61)*	1.26 (1.12, 1.41)*	1.42 (1.09, 1.85)*	Not performed	Not performed
*Subgroup analysis according to principal diagnosis*						
TBI	3 studies	3 studies	1 study	2 studies	1 study	1 study
Random-effects	1.21 (0.98, 1.51)	1.44 (0.91, 2.29)	1.05 (0.67, 1.66)	1.47 (1.16, 1.87)*	− 0.47 (− 0.82, − 0.12)*	− 0.47 (− 0.82, − 0.12)*
Fixed-effect	1.21 (0.98, 1.51)	1.44 (0.91, 2.29)	1.05 (0.67, 1.66)	1.47 (1.16, 1.87)*	− 0.47 (− 0.82, − 0.12)*	− 0.47 (− 0.82, − 0.12)*
CA	12 studies	12 studies	6 studies	6 studies	10 studies	7 studies
Random-effects	1.05 (0.96, 1.15)	1.13 (0.88, 1.47)	1.21 (1.03, 1.42)*	1.52 (0.95, 2.46)	0.12 (− 0.01, 0.25)	0.06 (− 0.02, 0.15)
Fixed-effect	1.04 (0.99, 1.10)	1.11 (0.95, 1.30)	1.24 (1.15, 1.33)*	1.36 (1.06, 1.76)*	0.09 (0.03, 0.16)*	0.06 (− 0.00, 0.13)
SAH	5 studies	5 studies	3 studies	3 studies	3 studies	4 studies
Random-effects	1.34 (1.14, 1.56)*	1.89 (1.33, 2.70)*	1.33 (1.03, 1.72)*	1.86 (0.91, 3.77)	0.40 (0.26, 0.53)*	0.68 (0.05, 1.30)*
Fixed-effect	1.31 (1.15, 1.48)*	1.83 (1.33, 2.50)*	1.32 (1.12, 1.54)*	1.61 (1.04, 2.50)*	0.40 (0.26, 0.53)*	0.52 (0.39, 0.64)*
Post-CPB	1 study	1 study	–	–	1 study	1 study
Random-effects	5.75 (0.35, 93.28)	6.60 (0.37, 117.31)	–	–	0.82 (0.18, 1.46)*	0.72 (0.08, 1.36)*
Fixed-effect	5.75 (0.35, 93.28)	6.60 (0.37, 117.31)	–	–	0.82 (0.18, 1.46)*	0.72 (0.08, 1.36)*
Ischemic stroke	1 study	1 study	1 study	1 study	–	–
Random-effects	1.41 (1.14, 1.74)*	2.03 (1.29, 3.21)*	1.47 (1.16, 1.85)*	2.27 (1.22, 4.23)*	–	–
Fixed-effect	1.41 (1.14, 1.74)*	2.03 (1.29, 3.21)*	1.47 (1.16, 1.85)*	2.27 (1.22, 4.23)*	–	–

Parameters in brackets are 95% CI

CA, cardiac arrest, CI, confidence interval, CPB, cardiopulmonary bypass, OR, odds ratio, PaO_2_, arterial partial pressure of oxygen, RR, relative risk, SAH, subarachnoid hemorrhage, SMD, standardized mean difference, TBI, traumatic brain injury

*Represent significant results

We performed the prespecified sensitivity analyses to test the robustness of the findings. By using a fixed-effect model, we again found a significant association between hyperoxemia and poor neurological outcome, and the OR agreed with the main finding (Table 3; Fig. 2a; Supplementary Fig. 1a). We also found that the association remained significant when restricting our analysis to good quality publications (RR 1.22, 95% CI 1.10–1.36, *p* = 0.007; Supplementary Fig. 2a), or when removing studies [39, 40] that introduced heterogeneity (RR 1.17, 95% CI 1.09–1.26, *p* < 0.001, *I*^2^ 33.7, *p* = 0.072 from Cochran Q test; Supplementary Fig. 2b). Post hoc sensitivity analysis using adjusted RR or adjusted OR supported the main findings (Table 3; Supplementary Fig. 1b–c).

Predefined subgroup analyses based on diseases revealed that hyperoxemia was significantly associated with poor neurological outcome in patients with SAH (RR 1.34, 95% CI 1.14–1.56, *p* < 0.001) and ischemic stroke (RR 1.41, 95% CI 1.14–1.74, *p* = 0.002, although only one study was included). The association was not significant in patients with CA (RR 1.05, 95% CI 0.96–1.15, *p* = 0.25), TBI (RR 1.21, 95% CI 0.98–1.51, *p* = 0.08), or post-CPB (RR 5.75, 95% CI 0.35–93.28, *p* = 0.21; Fig. 2a). Analyses according to disease using unadjusted and adjusted ORs and adjusted RRs are shown in Table 3 and Supplementary Fig. 1.

Hyperoxemia remained significantly associated with poor neurological outcome in post hoc subgroup analyses limited to functional outcomes (i.e., CPC, GOS, GOSE, and mRS) (Supplementary Fig. 3a). Grouping studies based on inclusion of hypoxemic patients in their controls, we found a significant association in studies without hypoxemia in controls but only a borderline significant association in studies including hypoxemia in controls (Supplementary Fig. 3b). Hyperoxemia was significantly associated with poor neurological outcome in a subgroup with studies using PaO_2_ cutoff points between 200 and 299 mm Hg, but neither with a PaO_2_ cutoff ≥ 300 mm Hg nor between 100 and 199 mm Hg (Supplementary Fig. 4a). Lastly, in subgroup analysis by ventilation status, hyperoxemia showed a nonsignificant trend toward poor neurological outcome in definitely ventilated patients and the unassessable ventilation status group, whereas a significant association was found in probably ventilated patients, and in mixed ventilated and nonventilated patients (Supplementary Fig. 4b). Unadjusted RRs of these analyses are shown in Supplementary file 7: Additional Table 1.

The post hoc meta-analysis to determine whether an increase in PaO_2_ leads to a greater odds of poor neurological outcome (on a natural log scale) showed a significant correlation (pooled slope 0.0024, 95% CI 0.0003–0.0045, *p* = 0.024; Supplementary Fig. 5; Supplementary file 7: Additional Table 2), with a substantial heterogeneity, when we considered all possible PaO_2_ levels including hypoxemia. This was equivalent to a 0.55% rise of odds of having poor neurological outcome when PaO_2_ is increased by 1 mm Hg. Comparable significant associations were found in sensitivity analyses using different methods of calculating standard error or using risk as an outcome (Supplementary file 7: Additional Table 2); however, the analysis using a categorical classification of oxygenation level showed a nonsignificant trend (Supplementary file 7: Additional Table 2). When hypoxemic levels of PaO_2_ were excluded, almost all associations from sensitivity analyses became significant (Supplementary file 7: Additional Table 2).

We determined the threshold PaO_2_ value which best differentiated poor and good neurological outcomes in patients with SAH, because they showed a robust association between hyperoxemia and poor neurological outcome. By taking every possible threshold from each study into account, we created a summary receiver operating characteristic curve (Supplementary Fig. 6a, b). The results show that the best differentiating threshold for PaO_2_ was 154 mm Hg with a pooled sensitivity of 57.9% (95% CI 38.5–75.2%) and a pooled specificity of 57.4% (95% CI 39.8–73.3%; Supplementary Fig. 6c, d).

### Secondary Objective

**Fig. 3:**
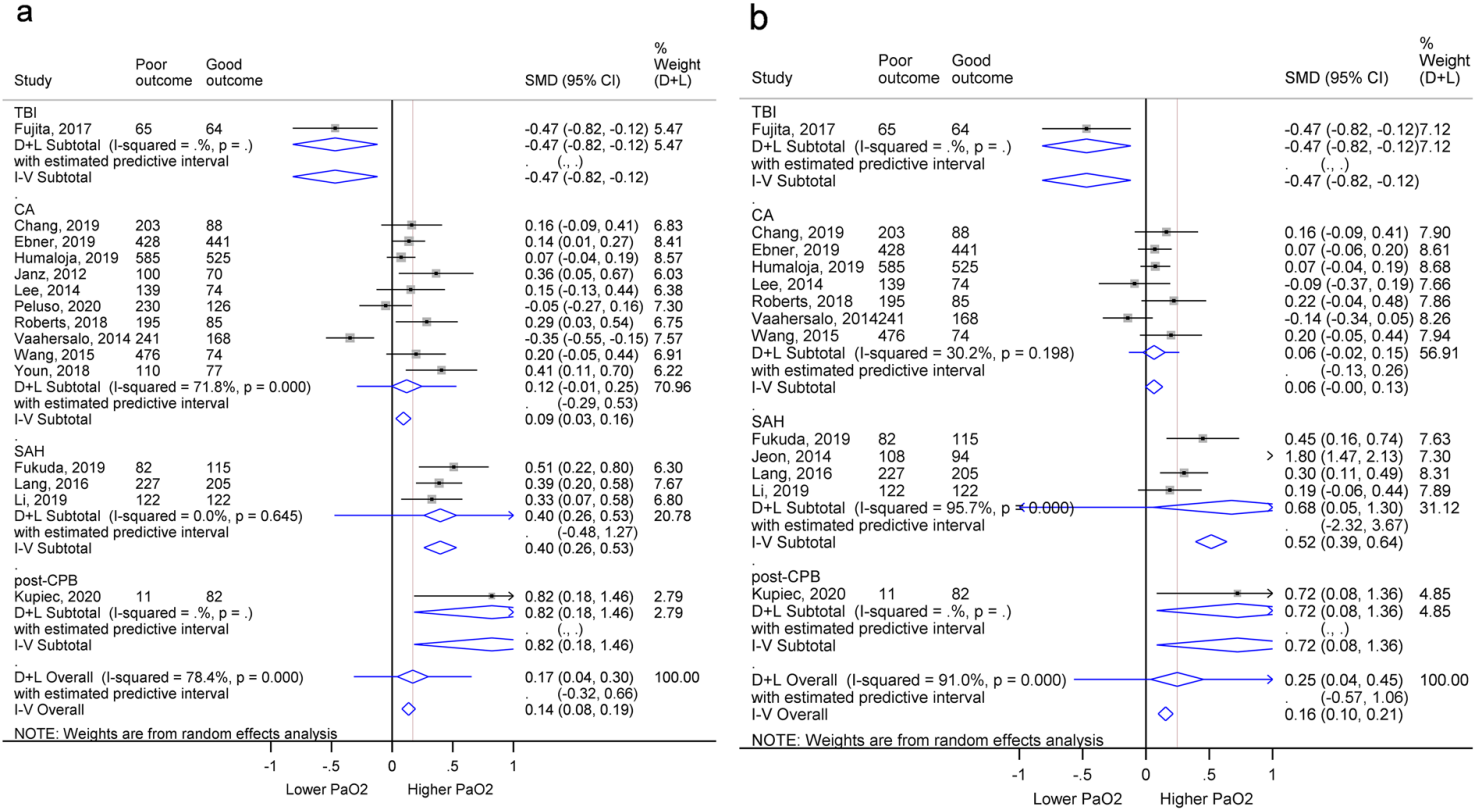
Forest plots comparing PaO_2_ in patients with poor and good neurological outcome.** a** Comparing maximum PaO_2_ values. **b** Comparing mean PaO_2_ values. The boxes show the effect estimates from the individual studies, and the diamonds represent pooled results in each subgroup and overall analysis. The length of horizontal lines across the boxes and the width of the diamonds illustrates the 95% CI. The gray vertical line at zero is the line of null effect and the red vertical line shows the pooled effect estimate of the whole analysis. CA, cardiac arrest, CI, confidence interval, CPB, cardiopulmonary bypass, PaO_2_, arterial oxygen partial pressure, SAH, subarachnoid hemorrhage, TBI, traumatic brain injury

We performed meta-analyses comparing PaO_2_ in patients with poor and good neurological outcomes. We used 15 studies, comprising 3214 poor outcome patients out of a total of 5530 for the secondary outcome analysis. This showed that the maximum PaO_2_ in the poor neurological outcome group was significantly higher than in patients with good neurological outcome (SMD 0.17, 95% CI 0.04–0.30, *p* = 0.046) with substantial heterogeneity (*I*^2^ 78.4%, *p* < 0.001; Fig. 3a). We then compared the mean PaO_2_ from 5019 patients, 2882 of which had poor neurological prognoses. Patients with poor outcome had a significantly higher mean PaO_2_ (SMD 0.25, 95% CI 0.04–0.45, *p* = 0.020) with significant heterogeneity (*I*^2^ 91.0%, *p* < 0.001; Fig. 3b). Further subgroup analyses of the maximum and mean PaO_2_ by diseases were performed, and the results are in Table 3.

## Discussion

### Main Findings

Our meta-analysis reveals two key points: (1) hyperoxemia was significantly associated with poorer neurological prognoses in patients with a range of acute illnesses (Figs. 2a, 3) with high robustness across all sensitivity analyses and types of outcome (categorical and continuous outcomes) and (2) there was a trend for poorer outcome in higher PaO_2_ groups, regardless of the inclusion of hypoxemia in controls (Supplementary Fig. 3b), PaO_2_ criteria used (Supplementary Figs. 4a, 5) or the ventilation status of the patients (Supplementary Fig. 4b).

In the subgroup analysis of different PaO_2_ cutoff points, a significant association was found only in one group (PaO_2_ cutoff values between 200 and 299 mm Hg; Supplementary Fig. 4a), but the lack of significance in the others might reflect small study numbers, decreasing statistical power. In the case of a PaO_2_ cutoff ≥ 300 mm Hg, another contributing factor is that a number of patients with poor neurological outcome with PaO_2_ < 300 mm Hg is added to the control group, resulting in a smaller effect size.

When studies were grouped according to ventilation status, the result became less precise, but all categories of ventilation status demonstrated a similar trend of poorer neurological prognoses in the hyperoxemia group (Supplementary Fig. 4b). The lack of significance might stem from a low number of studies in each category. There was a significant heterogeneity within some categories, suggesting that ventilation status was not the source of heterogeneity. Due to the uncertain nature of the ventilation status in the probably ventilated and the unassessable groups, results gained from these may not be as informative as from the ventilated group.

From the post hoc meta-analysis, we found a significant correlation between oxygenation level and poor neurological outcome with high robustness across sensitivity analyses (Supplementary Fig. 5). The result became more significant when we excluded defined hypoxemic levels from the analyses, suggesting that there might be a U-shaped association between PaO_2_ and poor neurological outcome, with both hypoxemia and hyperoxemia linked to worsened outcome. However, cautious interpretation is suggested because there was a high level of statistical heterogeneity, even though we pooled the data with a random-effects model.

**Fig. 4:**
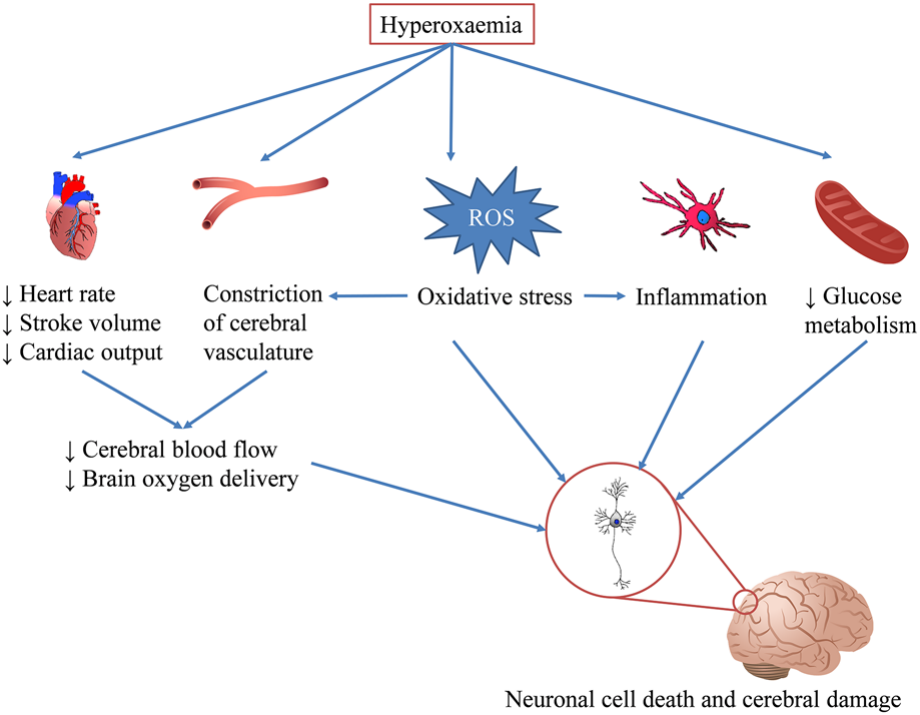
Effects of hyperoxemia on the brain. High oxygen causes constriction of the cerebral and the peripheral vasculature. As a result, blood pressure and cardiac afterload increase, triggering a reduction in heart rate, stroke volume, and cardiac output. Consequently, cerebral blood flow and oxygen delivery to the brain are decreased. Hyperoxemia also leads to oxidative stress, which can affect neurons and the brain directly and indirectly by promoting the constriction of cerebral vasculature via depletion of nitric oxide and stimulating inflammation. Furthermore, a high blood oxygen level might disturb glucose metabolism due to suppression of brain oxygen uptake. All of these effects result in neuronal death and cerebral damage. ROS, reactive oxygen species

The association of hyperoxemia and poor neurological outcomes correlates well with experimental evidence showing that high oxygen can be harmful to adult brains. In humans, despite the higher blood oxygen content in hyperoxemia, cerebral oxygen delivery can be reduced because of a lower blood flow [3], thus disrupting the energy supply to neurons. This decreased brain blood flow results from constriction of cerebral arterioles [41], large cerebral arteries [42] and conceivably capillary pericytes [43]. Additionally, hyperoxemia indirectly affects cerebral blood flow by reducing heart rate, stroke volume and cardiac output [3]. Hyperoxia might also depresses glucose metabolism in the brain [44] and increases oxidative stress [4], which both lead to cerebral damage (Fig. 4).

### SAH Outcomes

From our RR assessments, patients with SAH showed a robust association between hyperoxemia and unfavorable neurological outcome. Furthermore, patients with poor neurological outcome had significantly higher levels of PaO_2_ than patients with good outcome. Thus, the extensive neurological damage suffered by patients with SAH might be exacerbated by excessive oxygen administration.

Hyperoxemia has been suggested to affect the prognosis of patients with SAH in both the early brain injury (EBI) and delayed cerebral ischemia (DCI) phases. EBI occurs within 4 days as a direct effect of the aneurysm. DCI, including cerebral artery vasospasm and delayed cerebral infarction, starts at the fifth day after SAH [45]. During EBI, inflammation and constriction of the microcirculation occur [45], as also occur in hyperoxic preclinical studies [23, 41]. Thus, hyperoxemia may aggravate EBI [46]. In addition to the early stage of injuries, hyperoxemia, and the presence of oxidized hemoglobin during the DCI phase, can aggravate the cascade of events initiated during EBI [45] and cause constriction of major intracranial blood vessels [42], a key pathophysiological component of DCI [45]. Indeed, hyperoxemia is correlated with occurrence of DCI [46]. The similar effects of high oxygen and the pathological processes underlying SAH make it unsurprising that hyperoxemia worsens outcome in patients with SAH.

We found that 154 mm Hg is the threshold value of PaO_2_ that best differentiates poor and good neurological outcome groups in SAH, similar to the 150 mm Hg used by Lång et al. [47]. Although the sensitivity and specificity for predicting neurological outcome based on PaO_2_ were low compared with those of real diagnostic tests (Supplementary Fig. 6a, b), our finding is sufficient to emphasize that mild hyperoxemia might adversely affect neurological outcomes.

To our knowledge, no clinical trial studies have investigated the effect of hyperoxemia on neurological outcome in patients with SAH. Current guidelines do not consider oxygenation targeting in these patients [48], and thus a tighter regulation of PaO_2_ should be implemented in patients with SAH, as hyperoxemia potentially worsens their prognosis.

### CA Outcomes

In patients with CA, there was a trend, without reaching statistical significance, of favoring normoxemia over hyperoxemia to improve patients’ neurological outcome from postanoxic brain damage. Differences in the maximum and mean PaO_2_ in poor and good outcome patients were insignificant, with a trend toward a higher PaO_2_ in the unfavorable outcome group. Thus, high PaO_2_ may increase brain injury caused by cessation of the circulation, but the results should be interpreted cautiously because there were significant heterogeneities in all analyses of patients with CA.

Preclinical animal studies of CA showed a significant association of hyperoxia and worse neurological outcomes [23], reflecting increased oxidative stress and microcirculatory dysfunction in the brain caused by hyperoxemia after ROSC [49]. However, our study failed to show a significant correlation. This might be due to clinical and methodological diversity of the studies, which showed significant heterogeneity. Further investigation indicated that heterogeneity was mainly contributed by the studies of Kiguchi et al. [39] and Vaahersalo et al. [40]. Common features for only these two studies were that they only studied out-of-hospital CA (OHCA) participants and that hypoxemia was included in controls. Patients with OHCA often received delayed, lower quality treatment [50], thereby risking a longer duration of harmful hypoxia [51]. Furthermore, by including patients with hypoxemia as controls, the negative effects of hyperoxemia on neurological outcome might be masked by the more harmful effects of hypoxemia in the controls. After removing these two studies from the CA group, hyperoxemia was significantly associated with poor neurological outcome with low heterogeneity (Supplementary Fig. 2b). Analysis taking into account the location of CA and hypoxemia in controls might be needed to investigate this further.

Current guidelines for CA recommend using the highest available oxygen concentration after ROSC until arterial oxygen saturation or PaO_2_ can be measured, but that if oxyhemoglobin saturation is 100% then reducing oxygenation is suggested provided the saturation can be kept at ≥ 94% [51]. Although our results did not achieve significance, they showed a trend toward harm from hyperoxemia in patients post ROSC. Thus, we concur with the guideline not to maintain patients at an elevated oxygen level.

### TBI Outcomes

The relationship between hyperoxemia and poor neurological outcome was not significant in patients with TBI except that the adjusted OR showed a worsened outcome in the high PaO_2_ group. In contrast, the maximum and mean PaO_2_ were lower in the unfavorable outcome patients compared with favorable outcome patients (Fig. 3a, b). Thus, the effect of PaO_2_ on the brain function of patients with TBI is still inconclusive.

Studies on the cerebral effects of hyperoxia in patients with TBI are also conflicting. Aside from the direct assault to the brain, secondary brain injury from TBI causes neuronal death through excitotoxicity, mitochondrial dysfunction, changes in cerebral oxygen metabolism, oxidative stress and inflammation [52, 53]. Most of these mechanisms can be affected by hyperoxia, albeit in opposite ways. High oxygen can increase oxidative stress, which is detrimental [4] and might impair glucose oxidation after TBI [44]. However, others have found that hyperoxia improves metabolism in the injured brain [1]. In an injured area with impaired cerebral autoregulation, high inspired oxygen increases arterial oxygen content without vasoconstriction, so more oxygen reaches the tissue [3]. Across all the studies considered, the effect of hyperoxia was inconsistent, but some subgroups of patients with TBI have been suggested to benefit from hyperoxia [53]. More studies are needed to clarify this association. Because of inconclusive results, we suggest following a recent guideline recommending a PaO_2_ target of 80–120 mm Hg in patients with acute brain injury with or without clinically significant increased intracranial pressure [54] until more studies are performed.

### Other Conditions

Discussion of ischemic stroke, CPB and severe traumatic injuries, for each of which only one study was included, is given in Supplementary file 7: Additional File 7.

### Strengths and Limitations

To our knowledge, this is the first systematic consolidation of previous observational evidence, which suggested a potential negative effect of hyperoxemia on neurological outcomes, implying a negative impact on patients’ quality of life. Consistent results across various prespecified sensitivity analyses further strengthened the validity of our main findings.

A few limitations are worth noting. Firstly, our overall findings were affected by heterogeneity, due to variations in the cutoff values of hyperoxemia reported, the underlying conditions, timing of PaO_2_ assessment, and outcome measures used by each study. Nevertheless, subgroup analysis based on underlying conditions profoundly reduced the degree of heterogeneity, except in the studies of CA populations, suggesting that this issue is not a major concern. Secondly, our analysis was based on observational studies, therefore the observed association does not prove a causal relationship between hyperoxemia and worse neurological sequelae. The results are consistent with two interpretations: either hyperoxemia has negative consequences (as suggested by animal experiments showing that an elevated PaO_2_ level can lower oxygen delivery) or patients in the worst condition are given more oxygen (reverse causality). In addition, the poorer neurological outcome observed in patients with hyperoxemia might be confounded or modified by a poorer quality of care given to the patients—a factor that we cannot take into account in a study-level meta-analysis. Although publication bias cannot be excluded, we used Tweedie’s trim and fill method to estimate the result when publication bias was eliminated. In analyses for the second objective, we only included studies that acknowledged hyperoxemia so we would miss articles comparing only hypoxemia and normoxemia. Lastly, dichotomization of outcomes into poor and good might lead to loss of some information.

### Implications

#### Research Implications

We showed that individual diseases respond differently to hyperoxemia. For example, there was a strong association between high PaO_2_ and poorer neurological outcome in SAH but not in patients with TBI. This raises the question of whether oxygenation targets should be tailored to individual diseases and what PaO_2_ cutoff point to use for each disease. Support for the customization of oxygen therapy for each disease comes from the post hoc analysis of the intensive care unit randomized trial comparing two approaches to oxygen therapy (ICU-ROX) study, showing that conservative (minimal) oxygen therapy may cause harm (increased mortality) in patients with sepsis [55], while showing a possibility of benefit in patients with hypoxic ischemic encephalopathy [56] (although neither analysis reached significance). We attempted to determine the appropriate PaO_2_ threshold that best differentiates patients with SAH who experienced poor neurological outcome and good neurological outcome; however, a better approach might be to combine individual patient data from a large multicenter prospective or well-designed retrospective observational study so that we could treat PaO_2_ as a continuous variable to define the optimal threshold.

In addition, data from this meta-analysis could be used as a rationale for further clinical trials comparing a tighter oxygenation strategy, i.e., avoiding hyperoxemia and liberal oxygen usage. It would be valuable to test whether such a strategy is feasible in practice, and whether it leads to better neurological outcome, especially for SAH and ischemic stroke. It could also prove a causal relationship between hyperoxemia and worsened neurological outcome.

Lastly, this meta-analysis also highlights the absence of a consensus on the definition of hyperoxemia in terms of each type of PaO_2_ measured (e.g., highest PaO_2_, O_2_ burden, average PaO_2_), duration of hyperoxemia and cutoff values. Studies to determine these factors to define hyperoxemia might be needed to create a standard for comparing results across different studies.

### Clinical Implications

Our study, which is the first to draw conclusions on the relationship between hyperoxemia and neurological outcomes from multiple studies, highlights the possibility of adverse consequences of hyperoxemia in hospitalized patients, especially with SAH and ischemic stroke. This emphasizes the need for close monitoring of oxygenation, and titrating oxygen levels to target normoxemia, which might improve patients’ outcome. Although more studies are required, incorporating an optimal PaO_2_ level into SAH guidelines might be clinically helpful as it is easy to monitor and titrate PaO_2_ level in clinical practice. Moreover, because hyperoxemia was associated with poorer neurological outcome, hyperoxemia might be useful as a prognostic factor for patients’ neurological outcome and be helpful for patient counseling and preparation for discharge.

## Conclusions

Hyperoxemia is associated with unfavorable neurological outcome in adult patients with acute illnesses, especially for patients with SAH and ischemic stroke. Although it was still inconclusive for patients with CA, TBI, post-CPB and general trauma, no clear benefits of hyperoxemia for neurological outcome were detected for those patients. We hope that our data will be valuable in encouraging clinicians to monitor and correct the hyperoxemia commonly found in clinical practice. Further studies to investigate an optimal PaO_2_ cutoff point and clinical trials on the effects of tighter oxygen control, especially in patients with SAH, are required.

## Supplementary Information

Below is the link to the electronic supplementary material.

Supplementary file1 (TIF 1637 kb)

Supplementary file2 (TIF 1309 kb)

Supplementary file3 (TIF 1560 kb)

Supplementary file4 (TIF 1631 kb)

Supplementary file5 (TIF 384 kb)

Supplementary file6 (TIF 1959 kb)

Supplementary file7 (DOCX 106 kb)

## Abbreviations

CA: Cardiac arrest
CI: Confidence interval
CPB: Cardiopulmonary bypass
CPC: Cerebral Performance Category
EBI: Early brain injury
ECMO: Extra corporeal membrane oxygenation
FiO_2_: Fraction of inspired oxygen
DCI: Delayed cerebral ischaemia
GCS: Glasgow Coma Scale
GOS: Glasgow Outcome Scale
GOSE: Glasgow Outcome Scale extended
IQR: Interquartile range
mRS: Modified Rankin Scale
NOS: Newcastle-Ottawa Scale
OHCA: Out-of hospital cardiac arrest
OR: Odds ratio
PaO_2_: Arterial oxygen partial pressure
POD: Post-operative delirium
ROSC: Return of spontaneous circulation
RR: Relative risk
SAH: Subarachnoid haemorrhage
SD: Standard deviation
SMD: Standardised mean difference
SROC: Summary receiver operating characteristic
TBI: Traumatic brain injury
