# Supplementary figures and images for: The Effect of Hyperoxemia on Neurological Outcomes of Adult Patients: A Systematic Review and Meta-Analysis
Source: Neurocrit Care. 2022 Jan 31;36(3):1027–43. doi: 10.1007/s12028-021-01423-w (PMC9110471; doi:10.1007/s12028-021-01423-w)

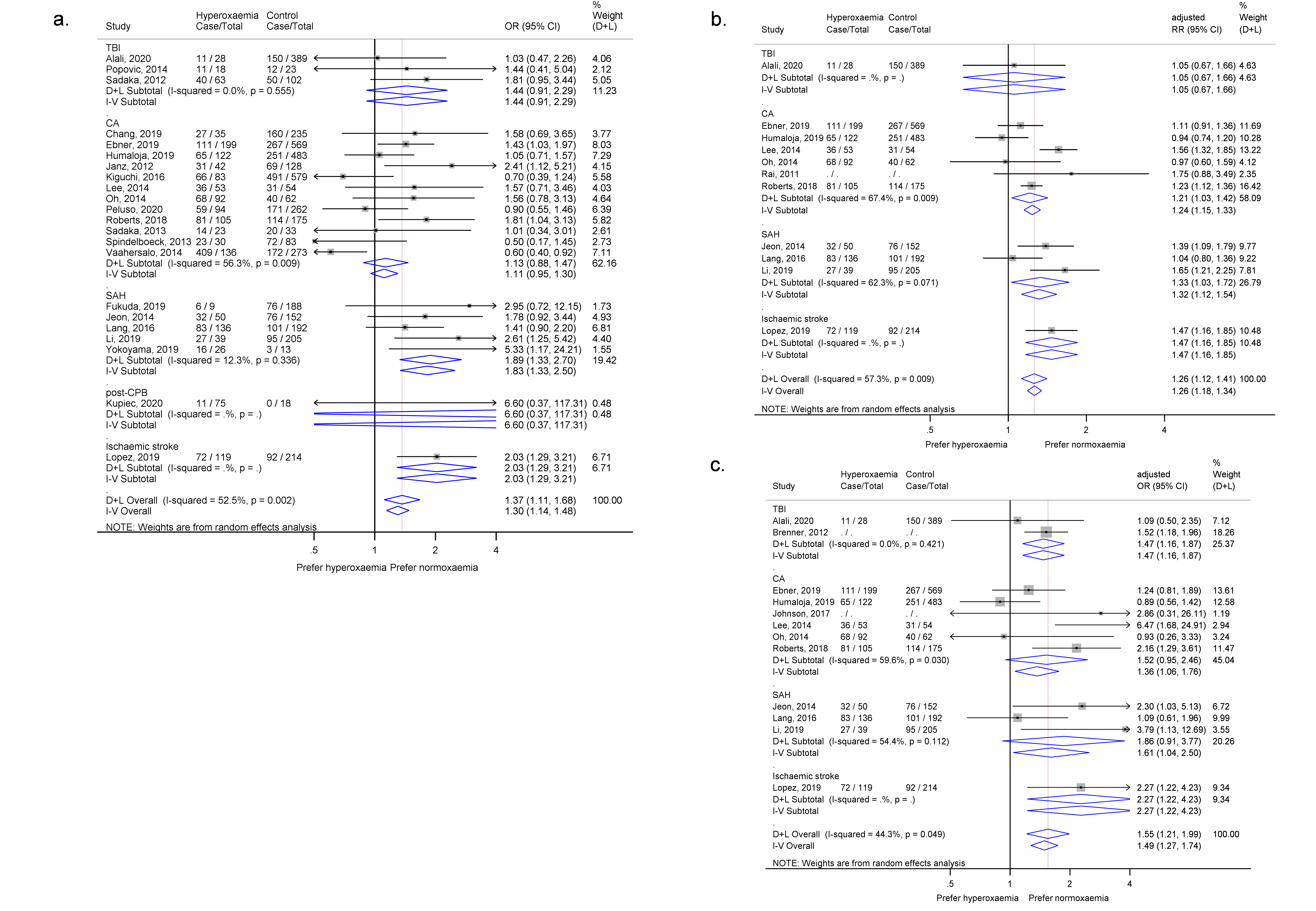

Supplement: Supplementary file 1 — Supplementary file1 (TIF 1637 kb) [file 12028_2021_1423_MOESM1_ESM.tif]

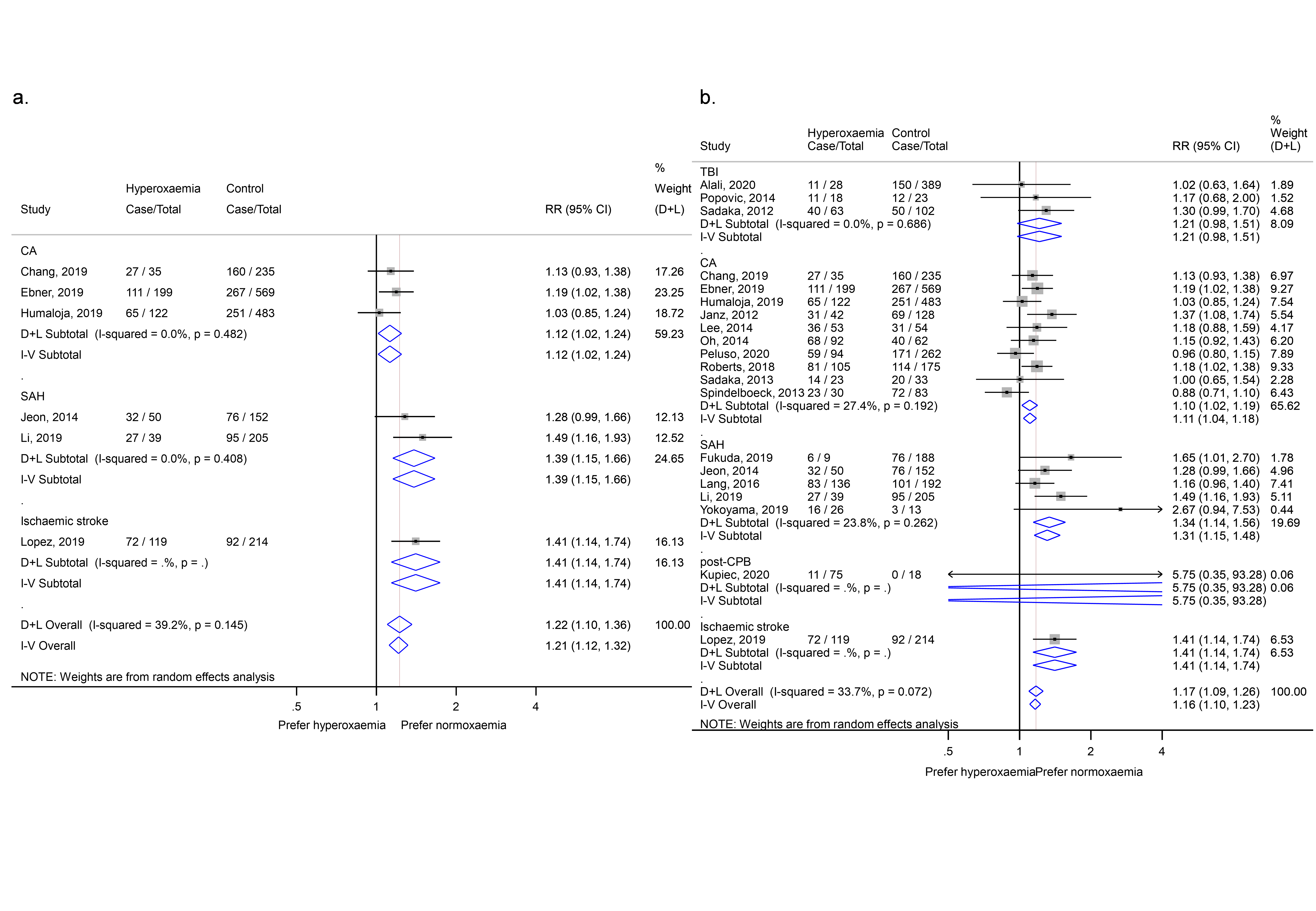

Supplement: Supplementary file 2 — Supplementary file2 (TIF 1309 kb) [file 12028_2021_1423_MOESM2_ESM.tif]

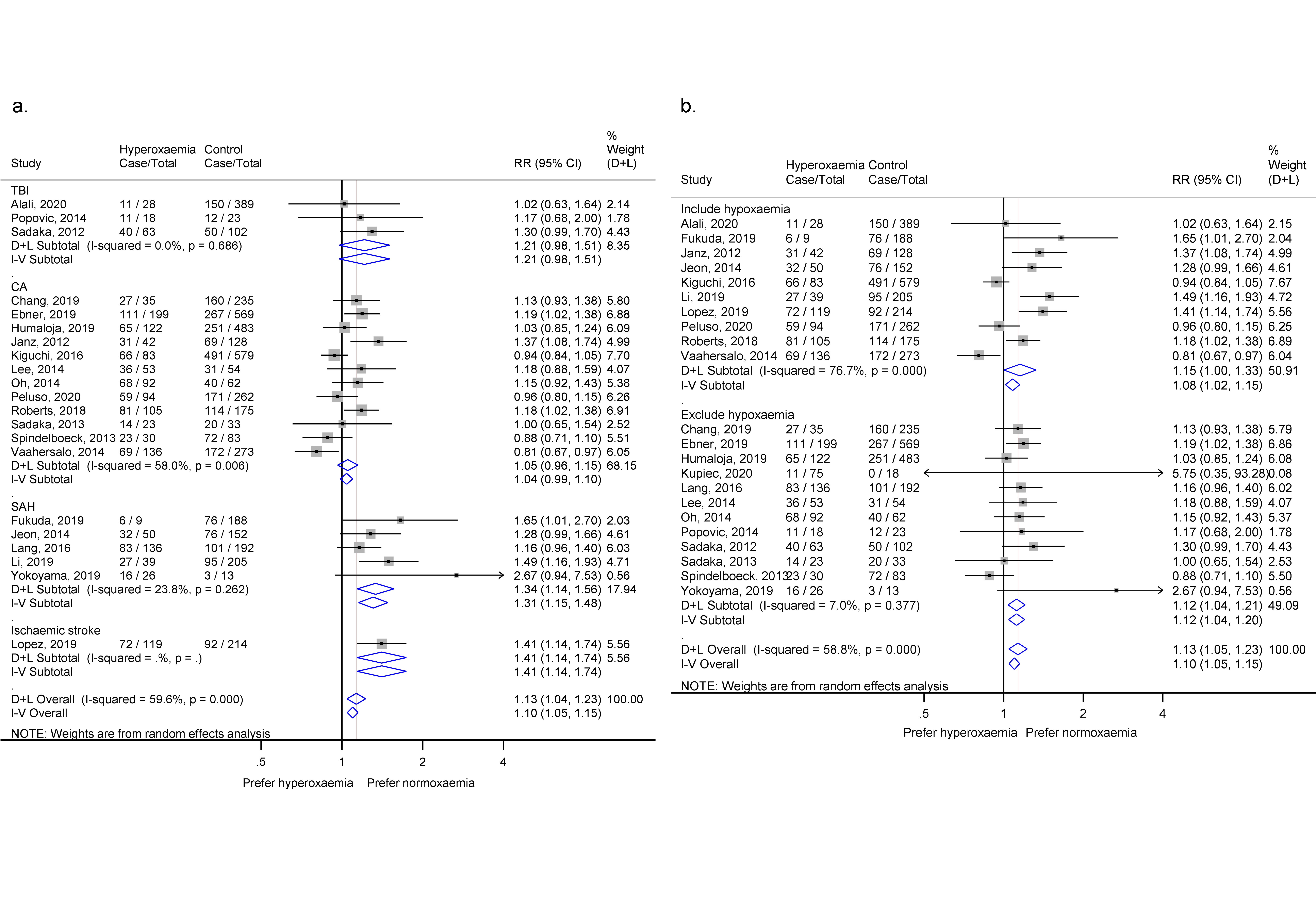

Supplement: Supplementary file 3 — Supplementary file3 (TIF 1560 kb) [file 12028_2021_1423_MOESM3_ESM.tif]

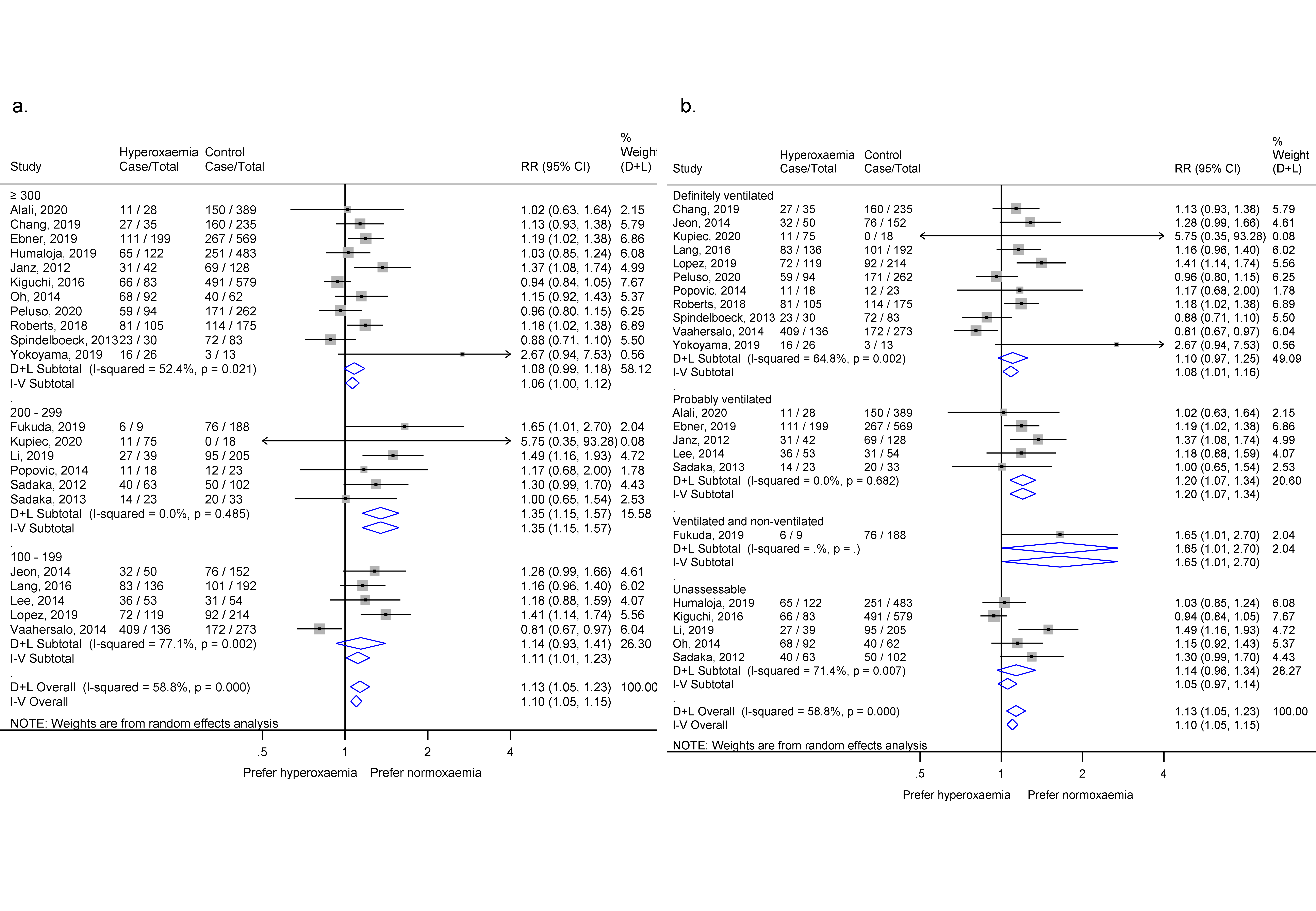

Supplement: Supplementary file 4 — Supplementary file4 (TIF 1631 kb) [file 12028_2021_1423_MOESM4_ESM.tif]

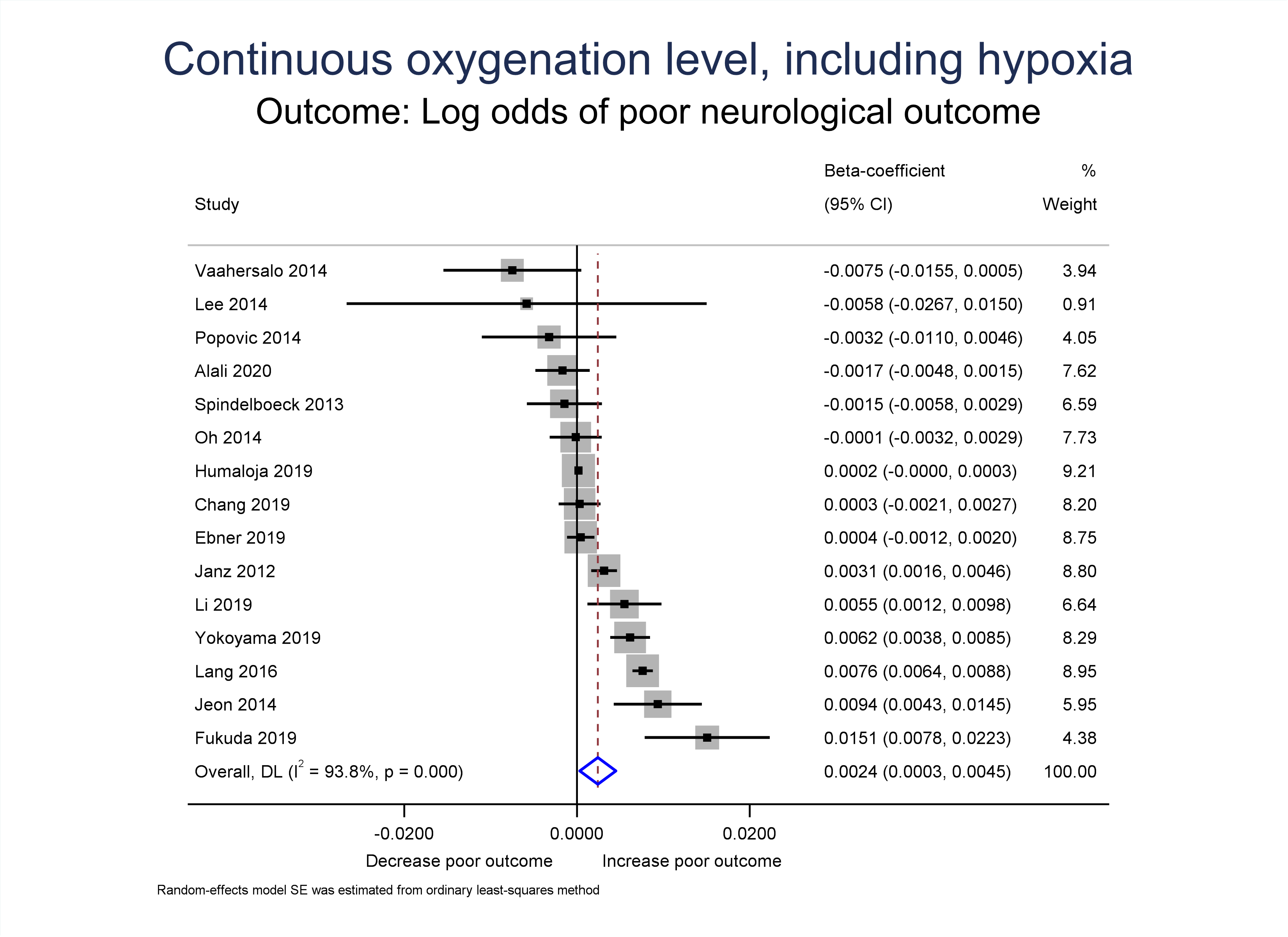

Supplement: Supplementary file 5 — Supplementary file5 (TIF 384 kb) [file 12028_2021_1423_MOESM5_ESM.tif]

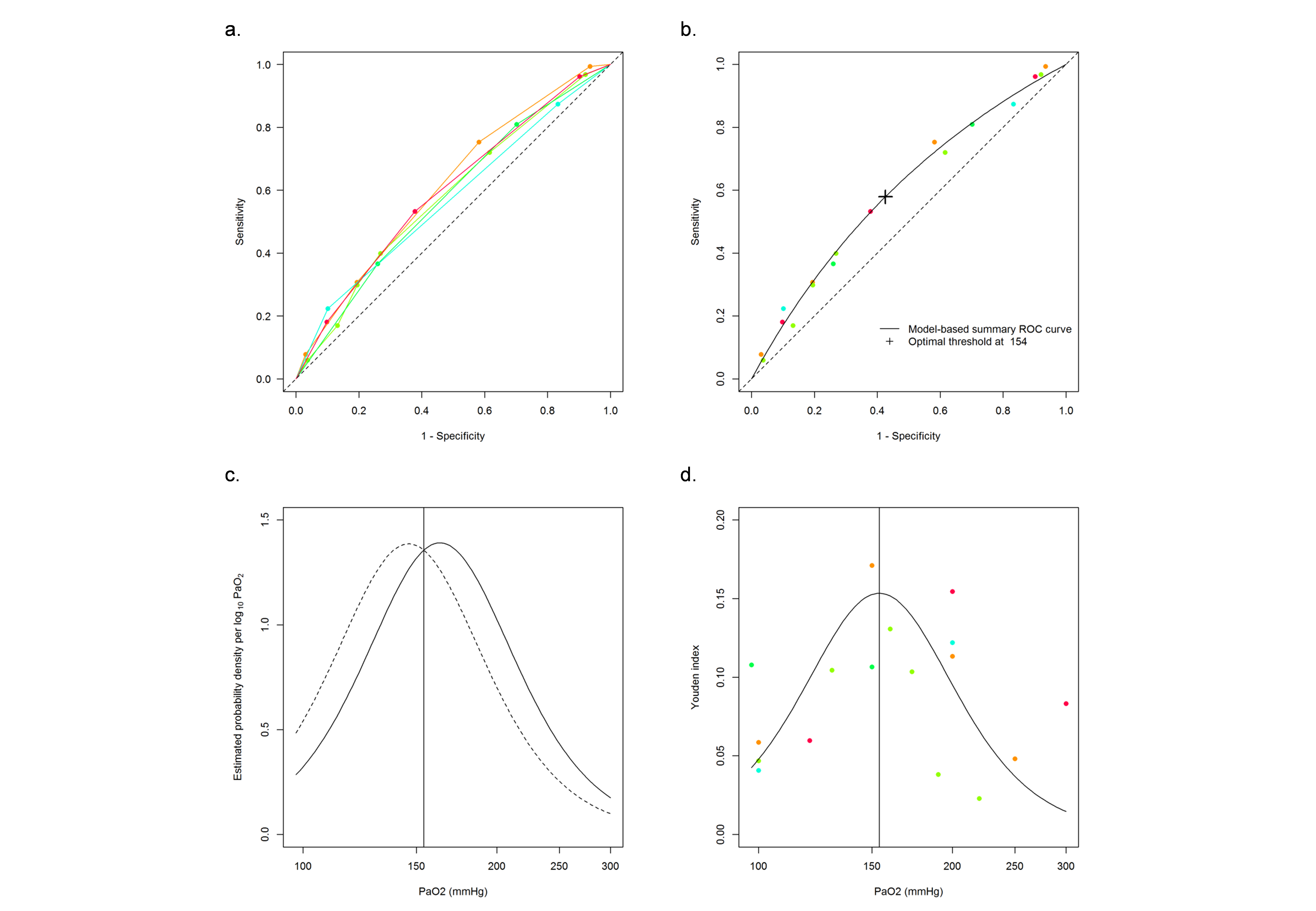

Supplement: Supplementary file 6 — Supplementary file6 (TIF 1959 kb) [file 12028_2021_1423_MOESM6_ESM.tif]
