# Supplementary material for: The Effect of Hyperoxemia on Neurological Outcomes of Adult Patients: A Systematic Review and Meta-Analysis
Source: Neurocrit Care. 2022 Jan 31;36(3):1027–43. doi: 10.1007/s12028-021-01423-w (PMC9110471; doi:10.1007/s12028-021-01423-w)
Supplement: Supplementary file 7 — Supplementary file7 (DOCX 106 kb) [file 12028_2021_1423_MOESM7_ESM.docx]

**Additional material**

Additional file 1. PRISMA checklist

Additional file 2. Keywords and search strategy

Additional file 3. Extracted information

Additional file 4. Method for meta-analysis to examine a correlation between oxygenation level and poor neurological outcome

Additional file 5. Method for finding a PaO_2_ threshold value to differentiate poor and good outcome groups

Additional file 6. Reasons for exclusion of 18 eligible articles

Additional file 7. Discussion on ischaemic stroke and other conditions

Additional table 1. Results for post-hoc subgroup analyses

Additional table 2. Results for meta-analysis of a correlation between oxygenation level and poor neurological outcome

Additional fig 1. Forest plots of sensitivity analyses using different study estimates for first objective.

Additional fig 2. Forest plots of sensitivity analyses limited to good quality studies and discarding sources of heterogeneity.

Additional fig 3. Forest plots of subgroup analyses limited to functional outcomes only and grouping by exclusion of hypoxaemia in controls.

Additional fig 4. Forest plots of subgroup analysis according to PaO_2_ cut-off value and ventilation status in each study.

Additional fig 5. Forest plots of post-hoc analysis on the correlation between oxygenation levels and poor neurological outcome

Additional fig 6. Post-hoc analysis of PaO_2_ threshold in subarachnoid haemorrhage patients that best differentiates poor and good outcomes

e-References

**Additional file 1. PRISMA Checklist**

| **Section/topic** | **Item No.** | **Checklist item** | **Reported on Page No.** |
| --- | --- | --- | --- |
| **Title** |  |  |  |
| Title | 1 | Identify the report as a systematic review, meta-analysis, or both | 1 |
| **Abstract** |  |  |  |
| Structured summary | 2 | Provide a structured summary including, as applicable, background, objectives, data sources, study eligibility criteria, participants, interventions, study appraisal and synthesis methods, results, limitations, conclusions and implications of key findings, systematic review registration number | 3-4 |
| **Introduction** |  |  |  |
| Rationale | 3 | Describe the rationale for the review in the context of what is already known | 5 |
| Objectives | 4 | Provide an explicit statement of questions being addressed with reference to participants, interventions, comparisons, outcomes, and study design (PICOS) | 5-6 |
| **Methods** |  |  |  |
| Protocol and registration | 5 | Indicate if a review protocol exists, if and where it can be accessed (such as web address), and, if available, provide registration information including registration number | PROSPERO (CRD42020187940) |
| Eligibility criteria | 6 | Specify study characteristics (such as PICOS, length of follow-up) and report characteristics (such as years considered, language, publication status) used as criteria for eligibility, giving rationale | 6-7 |
| Information sources | 7 | Describe all information sources (such as databases with dates of coverage, contact with study authors to identify additional studies) in the search and date last searched | 6 |
| Search | 8 | Present full electronic search strategy for at least one database, including any limits used, such that it could be repeated | 6, Additional file 2 |
| Study selection | 9 | State the process for selecting studies (that is, screening, eligibility, included in systematic review, and, if applicable, included in the meta-analysis) | 8 |
| Data collection process | 10 | Describe method of data extraction from reports (such as piloted forms, independently, in duplicate) and any processes for obtaining and confirming data from investigators | 8-9 |
| Data items | 11 | List and define all variables for which data were sought (such as PICOS, funding sources) and any assumptions and simplifications made | Additional file 3 |
| Risk of bias in individual studies | 12 | Describe methods used for assessing risk of bias of individual studies (including specification of whether this was done at the study or outcome level), and how this information is to be used in any data synthesis | 9 |
| Summary measures | 13 | State the principal summary measures (such as risk ratio, difference in means). | 9-10 |
| Synthesis of results | 14 | Describe the methods of handling data and combining results of studies, if done, including measures of consistency (such as I^2^) for each meta-analysis | 9-10 |
| Risk of bias across studies | 15 | Specify any assessment of risk of bias that may affect the cumulative evidence (such as publication bias, selective reporting within studies) | 9-10 |
| Additional analyses | 16 | Describe methods of additional analyses (such as sensitivity or subgroup analyses, meta-regression), if done, indicating which were pre-specified | 9-10, Additional file 4-5 |
| **Results** |  |  |  |
| Study selection | 17 | Give numbers of studies screened, assessed for eligibility, and included in the review, with reasons for exclusions at each stage, ideally with a flow diagram | 11, Figure 1, Additional file 6 |
| Study characteristics | 18 | For each study, present characteristics for which data were extracted (such as study size, PICOS, follow-up period) and provide the citations | 11-12, Table 1 |
| Risk of bias within studies | 19 | Present data on risk of bias of each study and, if available, any outcome-level assessment (see item 12). | 12, Table 2 |
| Results of individual studies | 20 | For all outcomes considered (benefits or harms), present for each study (a) simple summary data for each intervention group and (b) effect estimates and confidence intervals, ideally with a forest plot | Figure 2a-b, Figure 3 |
| Synthesis of results | 21 | Present results of each meta-analysis done, including confidence intervals and measures of consistency | 13-15, Table 3 |
| Risk of bias across studies | 22 | Present results of any assessment of risk of bias across studies (see item 15) | 13, Figure 2c |
| Additional analysis | 23 | Give results of additional analyses, if done (such as sensitivity or subgroup analyses, meta-regression [see item 16]) | 13-15, Additional table 1-2, Additional figure 1-6 |
| **Discussion** |  |  |  |
| Summary of evidence | 24 | Summarise the main findings including the strength of evidence for each main outcome; consider their relevance to key groups (such as health care providers, users, and policy makers) | 15-22, Additional file 7 |
| Limitations | 25 | Discuss limitations at study and outcome level (such as risk of bias), and at review level (such as incomplete retrieval of identified research, reporting bias) | 20 |
| Conclusions | 26 | Provide a general interpretation of the results in the context of other evidence, and implications for future research | 22 |
| **Funding** |  |  |  |
| Funding | 27 | Describe sources of funding for the systematic review and other support (such as supply of data) and role of funders for the systematic review | 24 |

**Additional file 2. Keywords and the search strategy**

**OVID (For Medline and Embase)**

(exp hyperoxia/ OR (hyperoxia* OR hyperoxic* OR hyperox?emi* OR normoxia* OR normoxic* OR normox?emi* OR hyperoxygenat* OR overoxygenat* OR ((high or supra*) ADJ2 (oxygen OR O2 OR PAO2 OR PO2 OR FIO2))) .tw.)

AND (outcome* OR disabilit* OR neurocogniti* OR cogniti* OR glasgow outcome OR GOS OR GOSE OR cerebral performance categor* OR CPC? OR rankin OR mRS OR barthel OR BI OR NIH* OR glasgow coma OR GCS) .tw.

AND (exp humans/ OR exp patient/ OR (human* OR patient* OR subject* ).tw.)

**Scopus**

TITLE-ABS-KEY (hyperoxia* OR hyperoxic* OR hyperoxemi* OR hyperoxaemi* OR normoxia* OR normoxic* OR normoxemi* OR normoxaemi* OR hyperoxygenat* OR overoxygenat* OR ((high OR supra*) PRE/2 (oxygen OR O2 OR PAO2 OR PO2 OR FIO2)))

AND TITLE-ABS-KEY (outcome* OR disabilit* OR neurocogniti* OR cogniti* OR “glasgow outcome” OR GOS OR GOSE OR “cerebral performance categor*” OR CPC OR CPCs OR rankin OR mRS OR barthel OR BI OR NIH* OR “glasgow coma” OR GCS)

AND TITLE-ABS-KEY (human OR humans OR patient OR patients OR subject OR subjects)

**Web of science**

TS = ((hyperoxia* OR hyperoxic* OR hyperox$emi* OR normoxia* OR normoxic* OR normox$emi* OR hyperoxygenat* OR overoxygenat* OR ((high OR supra*) NEAR/2 (oxygen OR O2 OR PAO2 OR PO2 OR FIO2)))

AND (outcome* OR disabilit* OR neurocogniti* OR cogniti* OR glasgow outcome OR GOS OR GOSE OR cerebral performance categor* OR CPC$ OR rankin OR mRS OR barthel OR BI OR NIH* OR glasgow coma OR GCS)

AND (human* OR patient* OR subject*))

**CINAHL**

(MH hyperoxia+ OR TX (hyperoxia* OR hyperoxic* OR hyperox#emi* OR normoxia* OR normoxic* OR normox#emi* OR hyperoxygenat* OR overoxygenat* OR ((high or supra*) N2 (oxygen OR O2 OR PAO2 OR PO2 OR FIO2))))

AND TX (outcome* OR disabilit* OR neurocogniti* OR cogniti* OR glasgow outcome OR GOS OR GOSE OR cerebral performance categor* OR CPC# OR rankin OR mRS OR barthel OR BI OR NIH* OR glasgow coma OR GCS)

AND (MH humans+ OR MH human+ OR MH patient+ OR TX (human* OR patient* OR subject*))

**ClinicalTrials.gov**

Condition or disease: hyperoxia OR hyperoxemia OR hyperoxaemia OR normoxia OR normoxemia OR normoxaemia

Intervention/treatment: hyperoxia OR hyperoxemia OR hyperoxaemia OR normoxia OR normoxemia OR normoxaemia

Study type: Observational studies

**Additional file 3. Extracted information**

The extracted information consisted of the following.

(a) Study detail: title, first author, publication year, corresponding author, study design.

(b) Population: principal diagnosis, additional data (e.g. therapeutic strategies), need for ventilation support, study eligibility criteria, overall age and gender, population size, number of cases.

(c) Exposure: types of PaO_2_ used (e.g. the highest/average/first PaO_2_), range of PaO_2_ in hyperoxaemic and control groups, duration of hyperoxaemia.

(d) Outcome measure: assessment criteria used for neurological outcomes.

(e) Results: number of patients in hyperoxaemic and control groups, with and without unfavourable outcomes, unadjusted and adjusted study estimates, adjusting factors, PaO_2_ in poor and good outcome groups.

**Additional file 4. Method for meta-analysis to examine a correlation between oxygenation level and poor neurological outcome**

For each study, we listed all PaO_2_ bands and the number of patients with poor and good neurological outcomes. We then calculated the midpoint of each PaO_2_ band as a representative for that particular PaO_2_ level. The odds of having poor neurological outcome for each PaO_2_ level were determined and the natural logarithm of odds (log (odds)) was calculated. We then linearly regressed log (odds) on the representative PaO_2_ for each study to find the slope of the line or beta-coefficient. A negative or a positive value of beta-coefficient indicated a decrease and an increase, respectively, in log (odds) of having a poor neurological outcome per one mmHg increase in PaO_2_. Finally, we pooled the beta-coefficients from individual studies using a random-effects model to yield an overall assessment of the relation between oxygenation level and the log (odds) of having a poor outcome.

Moreover, to ensure the robustness of the findings, we re-analysed the meta-regression of the plot of oxygen level against effect size (i.e. odds and risk) using various methods to calculate the standard error of the slope of the regression line (i.e. ordinary least-squares, robust, and bootstrapping). We also considered the dependence of log (odds) on oxygenation level using a categorical classification of oxygenation level i.e. the lowest PaO_2_ band was denoted 1, the second lowest was denoted 2, and so on. This approach did not rely on calculation of mid-points of the PaO_2_ levels so it would not be affected by the bias which might occur during the calculation (e.g. the mean of the PaO_2_ measurements within each PaO_2_ band might differ from the midpoint of the PaO_2_ band considered).

Additionally, we excluded a hypoxaemic mean PaO_2_ level (as defined by each individual study) to explore a potential U-shaped association.

**Additional file 5. Method for finding a PaO_2_ threshold value to differentiate poor and good outcome groups**

Further post-hoc analysis was performed to determine a PaO_2_ threshold value that best differentiated favourable and unfavourable outcome groups. We only analysed diseases that showed a significant robust association between PaO_2_ and poor neurological outcome. We adapted a method employed in a previous study [1] for meta-analysis of diagnostic tests by pooling the number of patients with poor and good outcomes, above and below all cut-off points from included studies, to create a summary receiver operating characteristic (SROC) curve. The threshold that gave the maximum Youden index [1] was chosen as the best estimate of the threshold value.

**Additional file 6. Reasons for exclusion of 18 eligible articles**

| **Study** | **Reason of exclusion** |
| --- | --- |
| Alali, 2018 [2] | Overlapping population with Alali, 2020 [3] |
| Belloni, 2013 [4] | Overlapping population with Peluso, 2020 [5] |
| Belloni, 2014 [6] | Overlapping population with Peluso, 2020 [5] |
| Ebner (a), 2019 [7] | Overlapping population with Ebner (b), 2019 [8] |
| Elmer, 2014 [9] | Overlapping population with Elmer, 2015 [10] |
| Fontes, 2014 [11] | Need clarification on exposure definition |
| Gaieski, 2012 [12] | Overlapping population with Johnson, 2017 (Penn Alliance for Therapeutic Hypothermia registry) [13] |
| Gaieski, 2013 [14] | Overlapping population with Johnson, 2017 (Penn Alliance for Therapeutic Hypothermia registry) [13] |
| Jeon, 2012 [15] | Overlapping population with Jeon, 2014 [16] |
| Lang, 2014 [17] | Overlapping population with Lang, 2016 [18] |
| Lee (a), 2014 [19] | Overlapping population with Lee (b), 2014 [20] |
| Li, 2018 [21] | Overlapping population with Li (a), 2019 [22] |
| Li (b), 2018 [23] | Overlapping population with Li (a), 2019 [22] |
| Peluso, 2018 [24] | Overlapping population with Peluso, 2020 [5] |
| Pollock, 2012 [25] | Overlapping population with Janz, 2012 [26] |
| Popovic, 2013 [27] | Overlapping population with Popovic, 2014 [28] |
| Rincon, 2020 [29] | Overlapping population with Alali, 2020 (Citicoline Brain Injury Treatment Trial) [3] |
| Shuker, 2014 [30] | Need clarification on outcome definition |

**Additional file 7. Discussion on ischaemic stroke and other conditions**

**Ischaemic stroke**

Although only one stroke study was assessed, we found a statistically significant association between hyperoxaemia and poor neurological outcome in all analyses (Fig. 2a and 3a-b). This suggests that excess oxygen administration may worsen the outcome of ischaemic brain insult.

Very few studies have investigated the effect of arterial hyperoxia on neurological status, and although the role of normobaric oxygen therapy (NBO) in ischaemic stroke has been studied in various clinical trials, the results were inconclusive [31]. Whilst not statistically significant, a recent meta-analysis of randomised control trials found that hyperoxia evoked a minor trend towards better short-term prognostic indicators (NIHSS), but worse long-term functional outcomes (Barthel index) and mortality [31].

The mechanism by which hyperoxaemia aggravates neuronal damage during ischaemic stroke may be through an increase in oxidative stress, which is a core pathological process in cerebral ischaemic-reperfusion injury [32]. The rate of reactive oxygen species production can be drastically elevated during reperfusion, resulting in lipid peroxidation, macromolecule cross-linking, endothelial cell damage and blood-brain barrier breakdown, inflammation and neuronal death [32]. Given that hyperoxaemia promotes further oxidative stress [33], it has been proposed to magnify neuronal injury [34].

Our results showing a significant association between hyperoxaemia and worsened neurological status are consistent with guidelines for acute ischaemic stroke contra-indicating oxygen supplementation in non-hypoxic patients [35].

**Other illnesses**

Only one study assessed neurological outcome (incidence of post-operative delirium) in post-CPB patients. Despite a trend, hyperoxaemia was insignificantly associated with poorer neurological outcome, possibly due to a small population size. The maximum and mean PaO_2_ were, however, significantly higher in subjects with unfavourable outcome compared to those with favourable outcome. These inconclusive results imply we need more studies to examine the effects of hyperoxaemia.

For severe traumatic injuries, only one study was included in the systematic review. No association between hyperoxaemia and neurological outcome was found. Similarly, a meta-analysis of trials on oxygen supplementation for trauma patients did not find any significant benefit or harm [36]. More studies are needed before reaching a conclusion.

**Additional table 1. Results for post-hoc subgroup analyses**

|  | **Unadjusted RR (95%CI) using random-effects model** | **p-value** |
| --- | --- | --- |
| - Limited to functional outcomes (i.e. CPC, GOS, GOSE and mRS) (21 studies) | **1.13 (1.04 – 1.23)** | **0.002** |
| - Subgroup analysis by exclusion of hypoxaemia in controls | | |
| Studies without hypoxaemia (12 studies) | **1.12 (1.04 – 1.21)** | **0.002** |
| Studies with hypoxaemia (10 studies) | **1.15 (1.00 – 1.33)** | **0.05** |
| - Subgroup analysis by PaO_2_ cut-off value | | |
| Studies with PaO_2_ cut-off ≥300 mmHg (11 studies) | 1.08 (0.99–1.18) | 0.089 |
| Studies with PaO_2_ cut-off points between 200-299 mmHg (6 studies) | **1.35 (1.15–1.57)** | **<0.001** |
| Studies with PaO_2_ cut-off points between 100-199 mmHg (5 studies) | 1.14 (0.93–1.41) | 0.219 |
| - Subgroup analysis by ventilation status | | |
| Studies with definitely ventilated patients (11 studies) | 1.10 (0.97–1.25) | 0.135 |
| Studies with probably ventilated patients (5 studies) | **1.20 (1.07–1.34)** | **0.001** |
| Studies with mixed ventilated and non-ventilated patients (1 study) | **1.65 (1.01-2.70)** | **0.047** |
| Studies with patients with unassessable ventilation status (5 studies) | 1.14 (0.96–1.34) | 0.135 |

Parameters in brackets are 95% CI. Results in bold represent significant results. CI: Confidence interval, CPC: cerebral performance category, GOS: Glasgow outcome score, GOSE: Glasgow outcome score – extended, mRS: Modified Rankin scale, RR: relative risk.

**Additional table 2. Results for meta-analysis of a correlation between oxygenation level and poor neurological outcome**

| **Analyses** | **Pooled beta-coefficient (95%CI) using random-effects model** | | | |
| --- | --- | --- | --- | --- |
|  | **Log odds of poor neurological outcome** | | **Log risk of poor neurological outcome** | |
|  | Per continuous increase in oxygenation level | Per categorical increase in oxygenation group | Per continuous increase in oxygenation level | Per categorical increase in oxygenation group |
| **Including hypoxaemia** |  |  |  |  |
| OLS SE | **0.0024**  **(0.0003, 0.0045)**  I^2^=93.8%, P<0.001 | 0.1501  (-0.0001, 0.3003)  I^2^=93.7%, P<0.001 | **0.0009**  **(0.0002, 0.0016)**  I^2^=87.2%, P<0.001 | **0.0627**  **(0.0023, 0.1230)**  I^2^=88.1%, P<0.001 |
| Robust SE | **0.0022**  **(0.0001, 0.0042)**  I^2^=96.5%, P<0.001 | 0.1356  (-0.0121, 0.2834)  I^2^=96.3%, P<0.001 | **0.0008**  **(0.0002, 0.0014)**  I^2^=92.5%, P<0.001 | 0.0588  (-0.0009, 0.1185)  I^2^=92.6%, P<0.001 |
| Bootstrapped SE^a^ | **0.0023**  **(0.0001, 0.0046)**  I^2^=88.9%, P<0.001 | 0.1536  (-0.0023, 0.3095)  I^2^=89.3%, P<0.001 | **0.0008**  **(0.0001, 0.0015)**  I^2^=75.6%, P<0.001 | 0.0565  (-0.0050, 0.1179)  I^2^=79.7%, P<0.001 |
| **Excluding hypoxaemia** |  |  |  |  |
| OLS SE | **0.0035**  **(0.0010, 0.0060)**  I^2^=89.5%, P<0.001 | **0.1919**  **(0.0251, 0.3586)**  I^2^=90.5%, P<0.001 | **0.0013**  **(0.0003, 0.0023)**  I^2^=86.3%, P<0.001 | **0.0760**  **(0.0090, 0.1430)**  I^2^=87.7%, P<0.001 |
| Robust SE | **0.0034**  **(0.0011, 0.0057)**  I^2^=92.4%, P<0.001 | **0.1889**  **(0.0239, 0.3539)**  I^2^=92.7%, P<0.001 | **0.0012**  **(0.0004, 0.0020)**  I^2^=91.0%, P<0.001 | **0.0749**  **(0.0105, 0.1394)**  I^2^=91.3%, P<0.001 |
| Bootstrapped SE^a^ | **0.0032**  **(0.0005, 0.0058)**  I^2^=76.4%, P<0.001 | **0.1783**  **(0.0141, 0.3424)**  I^2^=82.8%, P<0.001 | **0.0010**  **(0.0001, 0.0020)**  I^2^=70.5%, P<0.001 | 0.0605  (-0.0051, 0.1261)  I^2^=78.3%, P<0.001 |

^a^ Bootstrapping standard error with 500 replications, emboldened figure represents statistical significance.

Parameters in brackets are 95%CI. Results in bold represent significant results. CI: Confidence interval, OLS: Ordinary least-squares, SE: Standard error

**Additional fig 1. Forest plots of sensitivity analyses using different study estimates for first objective.**

(a) Using unadjusted OR. (b) Using adjusted RR. (c) Using adjusted OR. The boxes show the effect estimates from the individual studies and the diamonds represent pooled results in each subgroup and overall analysis. The length of horizontal lines across the boxes and the width of the diamonds illustrate the 95% CI. The grey vertical line at one is the line of null effect and the red vertical line shows the pooled effect estimate of the whole analysis.

**Additional fig 2. Forest plots of sensitivity analyses limited to good quality studies and discarding sources of heterogeneity.**

(a) Forest plot of unadjusted RRs of poor neurological outcome, limited to good quality studies. (b) Forest plots of unadjusted RRs of poor neurological outcome, discarding sources of heterogeneity. The boxes show the effect estimates from the individual studies and the diamonds represent pooled results in each subgroup and overall analysis. The length of horizontal lines across the boxes and the width of the diamonds illustrate the 95% CI. The grey vertical line at one is the line of null effect and the red vertical line shows the pooled effect estimate of the whole analysis.

**Additional fig 3. Forest plots of subgroup analyses limited to functional outcomes only and grouping by exclusion of hypoxaemia in controls**

(a) Forest plot of unadjusted RRs of poor neurological outcome, limited to functional outcomes only. (b) Forest plots of unadjusted RRs of poor neurological outcome, grouping by exclusion of hypoxaemia in controls. The boxes show the effect estimates from the individual studies and the diamonds represent pooled results in each subgroup and overall analysis. The length of horizontal lines across the boxes and the width of the diamonds illustrate the 95% CI. The grey vertical line at one is the line of null effect and the red vertical line shows the pooled effect estimate of the whole analysis.

**Additional fig 4. Forest plots of subgroup analysis according to PaO_2_ cut-off value and ventilation status in each study**

(a) Forest plot of unadjusted RRs of poor neurological outcome, grouping by PaO_2_ cut-off value. (b) Forest plots of unadjusted RRs of poor neurological outcome, grouping by patient ventilation status. The boxes show the effect estimates from the individual studies and the diamonds represent pooled results in each subgroup and overall analysis. The length of horizontal lines across the boxes and the width of the diamonds illustrate the 95% CI. The grey vertical line at one is the line of null effect and the red vertical line shows the pooled effect estimate for the whole analysis.

**Additional fig 5. Forest plots of post-hoc analysis on the correlation between oxygenation levels and poor neurological outcome**

Forest plot of beta-coefficients calculated from regression of log (odds) of having a poor neurological outcome on the midpoints of each PaO_2_ threshold in an individual study. The boxes show the effect estimates from the individual studies and the diamonds represent pooled results for the overall analysis. The length of horizontal lines across the boxes and the width of the diamonds illustrate the 95% CI. The grey vertical line at zero is the line of null effect and the red vertical line shows the pooled effect estimate of the whole analysis.

**Additional fig 6. Post-hoc analysis of PaO_2_ threshold in subarachnoid haemorrhage patients that best differentiates poor and good outcomes**

(a) ROC curves of individual studies assuming different PaO_2_ thresholds for defining good and poor outcomes. Each colour represents one study. Dashed black line represents a random chance of a good or poor outcome. Sensitivity is the probability of having hyperoxaemia in patients with poor neurological outcome, and specificity is the chance of not having hyperoxaemia in patients with good outcome. (b) Estimated SROC curve showing a threshold of PaO_2_ to differentiate patients with poor and good outcomes at 154 mmHg. Coloured points are as in (a). (c) Estimated probability densities (per log PaO_2_) of patients with poor neurological outcome (solid line) and good neurological outcome (dashed line) and their point of intersection. (d) Graph plotting Youden index (sensitivity + specificity – 1, y axis) against PaO_2_ (x-axis) with the threshold at 154 mmHg for maximization of the Youden index. Coloured points are as in (a).

**e-References**

1. Steinhauser S, Schumacher M, Rücker G. Modelling multiple thresholds in meta-analysis of diagnostic test accuracy studies. BMC Med Res Methodol 2016;16(1):1–15.

2. Alali A, Temkin NR, Vavilala M, et al. Matching resuscitation oxygenation to longterm outcome in severe traumatic brain injury: Target thresholds. J Neurotrauma [Internet] 2018;35(16):A-1-A-285. Available from: http://ovidsp.ovid.com/ovidweb.cgi?T=JS&PAGE=reference&D=emed19&NEWS=N&AN=623884239

3. Alali AS, Temkin N, Vavilala MS, et al. Matching early arterial oxygenation to long-term outcome in severe traumatic brain injury: target values. J Neurosurg 2020;132(2):537–44.

4. Belloni I, DellʼAnna A, Cortes DO, Vincent J-L, Taccone FS. Association between Paco2/Pao2 and neurological outcome after cardiac arrest. Crit Care Med [Internet] 2013 [cited 2020 Jun 22];41:A131. Available from: http://journals.lww.com/00003246-201312001-00497

5. Peluso L, Belloni I, Calabró L, et al. Oxygen and carbon dioxide levels in patients after cardiac arrest. Resuscitation 2020;150:1–7.

6. Belloni I, Dell’Anna A, Donadello K, Vincent J-L, Taccone FS. Arterial oxygen partial pressure in patients after cardiac arrest. Intensive Care Med [Internet] 2014;40(1 SUPPL. 1):S272. Available from: http://ovidsp.ovid.com/ovidweb.cgi?T=JS&PAGE=reference&D=emed15&NEWS=N&AN=71630795

7. Ebner F, Ullen S, Aneman A, et al. Associations between partial pressure of oxygen and neurological outcome in out-of-hospital cardiac arrest patients. Crit Care [Internet] 2019;23(S2):72. Available from: http://ovidsp.ovid.com/ovidweb.cgi?T=JS&PAGE=reference&D=emexa&NEWS=N&AN=627252744

8. Ebner F, Ullén S, Åneman A, et al. Associations between partial pressure of oxygen and neurological outcome in out-of-hospital cardiac arrest patients: an explorative analysis of a randomized trial. Crit Care 2019;23(1):30.

9. Elmer J, Pullalarevu R, Wang B, et al. The association between early hyperoxemia and oxygen exposure with development of pulmonary dysfunction, survival and neurological outcomes after cardiac arrest. Circulation [Internet] 2014;130(SUPPL. 2). Available from: http://ovidsp.ovid.com/ovidweb.cgi?T=JS&PAGE=reference&D=emed15&NEWS=N&AN=71709611

10. Elmer J, Scutella M, Pullalarevu R, et al. The association between hyperoxia and patient outcomes after cardiac arrest: analysis of a high-resolution database. Intensive Care Med 2015;41(1):49–57.

11. Fontes MT, McDonagh DL, Phillips-Bute B, et al. Arterial Hyperoxia During Cardiopulmonary Bypass and Postoperative Cognitive Dysfunction. J Cardiothorac Vasc Anesth [Internet] 2014 [cited 2020 Jun 22];28(3):462–6. Available from: https://linkinghub.elsevier.com/retrieve/pii/S105307701300178X

12. Gaieski DF, Grossestreuer AV, Perman SM, et al. Initial hyperoxia is associated with survival in postarrest patients enrolled in the PATH database. Circulation [Internet] 2012;126(21 SUPPL. 1). Available from: http://ovidsp.ovid.com/ovidweb.cgi?T=JS&PAGE=reference&D=emed13&NEWS=N&AN=70957143

13. Johnson NJ, Dodampahala K, Rosselot B, et al. The association between arterial oxygen tension and neurological outcome after cardiac arrest. Ther Hypothermia Temp Manag 2017;7(1):36–41.

14. Gaieski DF, Grossestreuer AV, Perman SM, et al. Initial hyperoxia is associated with survival in post-arrest patients enrolled in the path database. Acad Emerg Med [Internet] 2013;20(5 SUPPL. 1):S4–336. Available from: http://ovidsp.ovid.com/ovidweb.cgi?T=JS&PAGE=reference&D=emed14&NEWS=N&AN=71054173

15. Jeon S-B, Choi HA, Lantigua H, et al. Exposure to hyperoxia is associated with delayed cerebral ischemia and poor 3-month outcome after aneurysmal subarachnoid hemorrhage. Neurocrit Care [Internet] 2012;17(S2):1–337. Available from: http://ovidsp.ovid.com/ovidweb.cgi?T=JS&PAGE=reference&D=emed13&NEWS=N&AN=70930230

16. Jeon S-B, Choi HA, Badjatia N, et al. Hyperoxia may be related to delayed cerebral ischemia and poor outcome after subarachnoid haemorrhage. J Neurol Neurosurg Psychiatry 2014;85(12):1301–7.

17. Lang M, Raj R, Skrifvars M, et al. Association of blood gas tensions with outcome after acute subarachnoid hemorrhage. Intensive Care Med [Internet] 2014;40(1 SUPPL. 1):S120. Available from: http://ovidsp.ovid.com/ovidweb.cgi?T=JS&PAGE=reference&D=emed15&NEWS=N&AN=71630253

18. Lang M, Raj R, Skrifvars MB, et al. Early moderate hyperoxemia does not predict outcome after aneurysmal subarachnoid hemorrhage. Neurosurgery 2016;78(4):540–5.

19. Lee B, Jeung K, Jung Y, Lee S, Lee S. The relationship between time-weighted mean oxygen tension and outcome in out-of-hospital cardiac arrest survivors treated with therapeutic hypothermia. Resuscitation [Internet] 2014 [cited 2020 Jun 22];85:S95. Available from: https://linkinghub.elsevier.com/retrieve/pii/S0300957214003797

20. Lee BK, Jeung KW, Lee HY, et al. Association between mean arterial blood gas tension and outcome in cardiac arrest patients treated with therapeutic hypothermia. Am J Emerg Med 2014;32(1):55–60.

21. Li KC, Tam CWY, Shum HP, Yam WW. Impact of hyperoxia and hypocapnia on neurological outcome in patients with aneurysmal subarachnoid hemorrhage. Intensive Care Med Exp [Internet] 2018;6(S2):40. Available from: http://ovidsp.ovid.com/ovidweb.cgi?T=JS&PAGE=reference&D=emed19&NEWS=N&AN=624863567

22. Li KC, Tam CWY, Shum H-P, Yan WW. Impact of hyperoxia and hypocapnia on neurological outcomes in patients with aneurysmal subarachnoid hemorrhage: a retrospective study. Crit Care Res Pract 2019;2019:1–8.

23. Li KC, Tam CWY, Shum HP, Yan WW. Impact of hyperoxia and hypocapnia on neurological outcome in patients with aneurysmal subarachnoid haemorrha...20th Congress of Asia Pacific Association of Critical Care Medicine and Annual Scientific Meeting of Hong Kong SCCM 2018, 15-16 December 2018, . Crit Care Shock [Internet] 2019;22(1):63. Available from: http://search.ebscohost.com/login.aspx?direct=true&AuthType=ip,shib&db=jlh&AN=134448562&site=ehost-live&scope=site

24. Peluso L, Belloni I, Calabro L, Creteur J, Vincent J-L, Taccone FS. Oxygen and carbon dioxide in patients after cardiac arrest. Intensive Care Med Exp [Internet] 2018;6(S2):40. Available from: http://ovidsp.ovid.com/ovidweb.cgi?T=JS&PAGE=reference&D=emed19&NEWS=N&AN=624864805

25. Pollock J, Janz D, Hollenbeck R, McPherson J, Rice T. Hyperoxia is associated with increased mortality in patients treated with mild therapeutic hypothermia after cardiac arrest. Crit Care Med [Internet] 2012;40(12 SUPPL. 1):1–328. Available from: http://ovidsp.ovid.com/ovidweb.cgi?T=JS&PAGE=reference&D=emed13&NEWS=N&AN=71065671

26. Janz DR, Hollenbeck RD, Pollock JS, McPherson JA, Rice TW. Hyperoxia is associated with increased mortality in patients treated with mild therapeutic hypothermia after sudden cardiac arrest. Crit Care Med 2012;40(12):3135–9.

27. Popovic V, Pelcl T, Spindler M, Ketis Z, Strnad M. Prehospital hyperoxemia does not influence the functional neurological outcome in polytraumatized patients with traumatic head injury. Crit Care [Internet] 2013 [cited 2020 Jun 22];17(Suppl 2):P285. Available from: http://ccforum.biomedcentral.com/articles/10.1186/cc12223

28. Popović VV, Lesjak VB, Pelcl T, Strnad M. Impact of pre-hospital oxygenation and ventilation status on outcome in patients with isolated severe traumatic brain injury. Signa Vitae 2014;9(1):42.

29. Rincon F, Serruya M, Jallo J. Arterial Hyperoxia Is Associated with Poor Functional Outcome and Cognitive Impairment After TBI: A Retrospective Multi-center Cohort Study (1272). Neurology [Internet] 2020;94(15 Supplement):1272. Available from: http://n.neurology.org/content/94/15_Supplement/1272.abstract

30. Shuker BA, Bassford CR. Hyperoxaemia is associated with increased all-cause intensive care unit mortality in spontaneous subarachnoid haemorrhage. Intensive Care Med [Internet] 2014;40(1 SUPPL. 1):S244–5. Available from: http://ovidsp.ovid.com/ovidweb.cgi?T=JS&PAGE=reference&D=emed15&NEWS=N&AN=71630695

31. Ding J, Zhou D, Sui M, et al. The effect of normobaric oxygen in patients with acute stroke: a systematic review and meta-analysis. Neurol Res 2018;40(6):433–44.

32. Wu L, Xiong X, Wu X, et al. Targeting oxidative stress and inflammation to prevent ischemia-reperfusion injury. Front Mol Neurosci [Internet] 2020;13. Available from: https://doi.org/10.3389/fnmol.2020.00028

33. Mattos JD, Campos MO, Rocha MP, et al. Human brain blood flow and metabolism during isocapnic hyperoxia: the role of reactive oxygen species. J Physiol 2019;597(3):741–55.

34. López HV, Vivas MF, Ruiz RN, et al. Association between post-procedural hyperoxia and poor functional outcome after mechanical thrombectomy for ischemic stroke: an observational study. Ann Intensive Care 2019;9(1):59.

35. Powers WJ, Rabinstein AA, Ackerson T, et al. Guidelines for the early management of patients with acute ischemic stroke: 2019 update to the 2018 guidelines for the early management of acute ischemic stroke a guideline for healthcare professionals from the American Heart Association/American Stroke A. Stroke 2019;50(12):E344–418.

36. Grodum T, Stokholm Baekgaard J, Steinmetz J, Rasmussen LS. Initial use of supplementary oxygen for trauma patients: a systematic review. BMJ Open 2018;8:20880.
